# Dynamics of droplet breakup symmetrically placed between two collapsing cavities via numerical simulations

**DOI:** 10.1016/j.ultsonch.2025.107493

**Published:** 2025-08-05

**Authors:** Deepak K. Pandey, Rupak Kumar, Vivek V. Ranade

**Affiliations:** Multiphase Reactors and Intensification Group, Bernal Institute, University of Limerick, Limerick V94T9PX, Ireland

**Keywords:** Viscosity ratio, Interfacial tension, Ratio of cavity to droplet size, Driving pressure, Energy dissipation rate

## Abstract

Hydrodynamic cavitation is increasingly used for the production of liquid–liquid emulsions, yet the detailed mechanisms of droplet breakup induced by cavity collapse remain poorly understood. This study presents direct numerical simulations (DNS) of oil droplet fragmentation under the influence of two symmetrically collapsing cavities in water, mimicking conditions in cavitation-based emulsification devices. A volume-of-fluid (VOF) multiphase model is employed to examine the effects of interfacial tension (σ), viscosity ratio (λ), droplet-to-cavity size ratio (β), and driving pressure (ΔP) on droplet deformation and energy dissipation rate (ε). Unlike prior studies focused on single-cavity interactions or turbulent flows, this work reveals that symmetric cavity collapse generates complex, multi-phase breakup dynamics involving vortex-induced deformation and secondary droplet formation. Results indicate that ε increases with an increase in β, $\sigma_{\mathrm{dc}}$ and ΔP, whereas higher values of λ result in a decrease in ε. The dimensionless droplet perimeter $\left(P/P_{0}\right)$ was found to vary exponentially with the key parameters. The dimensionless perimeter of the droplet at the time of breakup $\left(P_{B}\right)$ decreases with an increase in $\sigma_{\mathrm{dc}}$, λ, ΔP and increases with β. A quantitative relationship is proposed between energy dissipation rate (ε), key parameters and dimensionless numbers (Weber and Ohnesorge numbers), identifying driving pressure and interfacial tension as dominant contributors. These insights enhance the mechanistic understanding of cavitation-driven emulsification and offer a foundation for optimising droplet size control and energy efficiency in industrial cavitation systems.

## Nomenclature

a: Constant, −
b: Constant, −
B: Empirical parameter, MPa
c: Constant, −
C: Fitting parameter, N
d: Droplet, −
k: Exponential rate constant, $\mu s^{-1}$
L: Initial distance between the cavity surface and the oil droplet surface, μm
N: Adiabatic exponent, −
P: Perimeter,μm
R: Radius, μm
t: Time,μs
T: Temperature, K
U, *u*: Velocity, m/s
V: Volume of phase, m^3^

**Greek letters**
α: Volume fraction, −
β: Size ratio, −
ρ: Density, kg/m^3^
μ: Dynamic viscosity, Pa.s
∇.: Divergence operator, −
∇: Gradient operator, −
ΔP: Driving Pressure, Pa
κ: Surface curvature, m^−1^
γ: Stand-off distance, −
σ: Interfacial tension, N/m
*ε*: Energy dissipation rate, m^2^/s^3^
λ: Viscosity ratio, −
$\dot{\gamma }$: Strain rate, s^−1^
$\varepsilon^{*}$: Non-dimensional energy dissipation rate, −
$\varepsilon_{o}$: *ε* generated by collapse of a single cavity in an infinite fluid domain without presence of any object in its vicinity, m^2^/s^3^
δ: Dirac delta function, 1/m
φ: Stand-off parameter when $R_{d}\to \infty$, −
τ: Time,μs

**Subscript**
B: Breakup
c: Continuous medium
cav: Cavity
d: Droplet
*i, j*: Phase pairs
init, 0: Initial
o: Reference

**Acronyms**
FVM: Finite Volume Method
DSD: Droplet size distribution
CFD: Computational Fluid Dynamics
CSF: Continuum surface force model
DNS: Direct Numerical Simulation
VOF: Volume of Fluid
EoS: Equation of State
BIM: Boundary Integral Method
LBM: Lattice Boltzmann Method
LES: Large Eddy Simulation
PIMPLE: Pressure-Implicit Method for Pressure-Linked Equations
MULES: Multidimensional universal limiter with explicit solution
NCG: Non-condensable gas
We: Weber number
Ca: Capillary number
Oh: Ohnesorge number

## 1 Introduction

Emulsions, particularly liquid–liquid emulsions, have a wide range of applications across various industries such as healthcare, food and nutrition, personal care, agrochemicals, and home care. In recent years, cavitation-based techniques for producing emulsions have gained considerable attention. Among these, hydrodynamic cavitation stands out as a particularly effective method [1,2]. Various fluidic devices leveraging hydrodynamic cavitation have been developed for emulsion generation [[3], [4], [5]]. To effectively simulate the emulsification process and predict droplet size distribution (DSD) in such systems, robust droplet breakage models are essential. A key factor in these models is the precise estimation of the energy dissipation rate (ε) within the emulsification system. Prior research has explored the correlation between droplet breakage and ε in turbulent flow environments [[6], [7], [8], [9]], where ε significantly influences droplet deformation and fragmentation. However, most of the existing turbulent breakage models rely on ε predicted through conventional turbulence models, which are not equipped to capture the highly localized and intense energy dissipation that occurs during the collapse of cavities in hydrodynamic cavitation-based systems. Pandey et al. [10] investigated how energy dissipation generated by a collapsing cavity affects a nearby deformable droplet, considering various configurations of size ratios and stand-off distances. Their findings indicate that despite the presence of intense localized energy, droplet breakup may not occur if the collapse-induced jet is oriented away from the droplet. In practical cavitation systems, multiple collapsing cavities and the associated flow fields interact to cause droplet breakup [9], underscoring the need for a more detailed understanding of the underlying breakup mechanisms driven by cavity collapse. This study seeks to advance fundamental insights in this domain, which are essential for the development of accurate and predictive models of cavitation-induced droplet breakage.

The theoretical foundation for turbulent droplet breakup dates back to the seminal contributions of Kolmogorov [11] and Hinze [12], who characterized the phenomenon as a balance between turbulent stresses and stabilizing forces such as surface tension. Building upon this framework, Walstra [13] introduced the concepts of turbulent inertia and viscous regimes, characterized by the Weber and Capillary numbers. Subsequent refinements by Davies [14] and Calabrese et al. [15] highlighted the stabilizing influence of internal rotational flows, especially at high viscosity ratios. Wang and Calabrese [16] further explored the combined effects of viscosity and interfacial tension, while Walstra and Smulders [17] emphasised the critical role of interaction time between droplets and turbulent eddies. More recent studies have deepened our understanding of these phenomena. Håkansson and Nilsson [18] demonstrated the influence of emulsifier adsorption kinetics on droplet deformation and breakup under turbulent conditions. Zhong and Ni [19] conducted a comprehensive evaluation of experimental data on breakup frequencies, highlighting the importance of characteristic timescales and phase properties. Experimental investigations into droplet breakup have been widely reported [7,[20], [21], [22], [23], [24], [25], [26]], with ε emerging as a critical parameter due to its appearance in key dimensionless numbers such as the Weber (We), Capillary (Ca), and Ohnesorge (Oh) numbers. These numbers govern the behaviour of droplets under various flow conditions [27,28]. However, accurately measuring ε in experiments remains challenging, primarily due to limitations in spatial and temporal resolutions of measurement techniques. This limitation necessitates the use of complementary approaches such as high-fidelity computational fluid dynamics (CFD) to study droplet–turbulence interactions. In this context, Sun et al. [29] employed large eddy simulations (LES) with adaptive mesh refinement to resolve fine-scale droplet–turbulence interactions. Li et al. [30] examined the secondary breakup behaviour of shear-thinning droplets, showing how non-Newtonian fluid properties can alter breakup dynamics. However, these studies have predominantly focused on breakups induced by turbulent shear flows [31], leaving the mechanisms of cavitation-induced breakup largely unexplored.

Several investigations have explored the interactions between the cavity and the droplet. Ohl et. al [32] examined the effect of a thin oil film positioned between the cavity and a solid boundary. Their results indicated that the presence of this oil layer had no significant impact on the direction of the cavity jet. Raman et al. [33] conducted both numerical simulations and experimental studies to analyze the interaction between a cavity and a water droplet immersed in a continuous silicone oil medium, focusing particularly on the influence of fluid viscosity. Their findings revealed that in highly viscous media, the collapsing cavity behaves like a flow sink, elongating and drawing the droplet toward it, eventually resulting in the formation of a droplet jet and the generation of secondary water droplets. Conversely, in media with lower viscosity, the water droplet tends to encapsulate the collapsing cavity, undergoing repeated cycles of expansion and collapse, which can lead to the formation of small oil droplets within the water droplet. Yamamoto and Komarov [34] investigated cavity collapse near oil droplets suspended in water, where cavities were generated using acoustic cavitation. They studied how the spatial separation between the cavity and the droplet affects the collapse dynamics and observed that the cavity jet is consistently directed away from the oil droplet toward the water. Furthermore, the velocity of the cavity jet was found to be dependent on the initial cavity-to-droplet distance. Despite these valuable insights, existing studies have primarily emphasized breakup phenomena driven by turbulent shear flows [31] and cavitation-induced fragmentation, with limited attention given to systematically analyzing the key parameters influencing cavitation-induced breakup mechanisms.

The literature indicates that most studies on droplet deformation and fragmentation have been centred on turbulent flow or collapsing cavity conditions, with limited investigation into the parametric effects of collapsing cavities. Understanding the mechanism of droplet breakup driven by cavity collapse is critical for advancing hydrodynamic cavitation as an emulsification technique. Such insights are particularly important for the rational design and optimisation of cavitation-based fluidic devices. In this study, we investigate the breakup of oil droplets in the vicinity of collapsing cavities using DNS. The influence of key parameters, including interfacial tension, droplet viscosity, size ratios, and initial cavity pressure, on energy dissipation and droplet breakup behaviour is systematically analyzed. The energy dissipation rates generated during cavity collapse are computed for each configuration. Sections 2, 3, and 4 of this paper detail the theoretical framework, numerical methodology, grid independence assessment, model validation, and the results pertaining to droplet breakup. The findings of this study provide a foundational basis for the development of advanced droplet breakage models tailored for hydrodynamic cavitation systems.

## 2 Theoretical background

Droplet deformation and eventual breakup result from an imbalance between external forces acting on the droplet and the internal resistance provided by σ. When exposed to external flow, the droplet undergoes deformation characterized by the strain rate $\dot{\left(\gamma \right)}$ and is given by:

(1) $$\dot{\gamma }=\frac{U_{dc}}{d_{d}}=\frac{\left(U_{d}-U_{c}\right)}{d_{d}}$$

Where, $U_{dc}$ is the relative velocity between the droplet and the continuous medium, $U_{d}$ and $U_{c}$ are the velocities of the droplet and the continuous medium, and $d_{d}$ is the diameter of the droplet.

The relative magnitudes of inertial force causing deformation versus the surface force resisting it are characterised using the We, which is defined as:

(2) $$We=\frac{Inertial\;forces}{Surface\;force}=\frac{\rho_{d}{U_{dc}}^{2}d_{d}}{\sigma_{dc}}$$

Where, $\rho_{d}$ is the density of the droplet and $\sigma_{dc}$ is the interfacial tension between the droplet and the continuous fluid. There is a threshold value of We above which the droplet cannot withstand the external forces and breaks. This threshold is called the critical Weber number (${We}_{crit})$ ​. If $We<{We}_{crit}$, the droplet remains intact whereas for $We>{We}_{crit}$​, drop breakup occurs.

In scenarios where viscosity significantly influences droplet deformation or breakup, particularly under high flow conditions, Oh becomes important [3]. The Oh characterizes the relative importance of viscous forces compared to inertial and interfacial forces and is defined as:

(3) $$Oh=\frac{\mu_{d}}{\sqrt{\rho_{d}\;\sigma_{dc}\;d_{d}}}$$

Where, $\mu_{d}$ is the dynamic viscosity of the droplet fluid. A higher Oh value indicates that viscous effects dominate over inertial and capillary effects, thereby playing a key role in the droplet’s deformation and stability. To include the effect of viscosity of both phases, the λ is relevant [35], which is defined as:

(4) $$\lambda =\frac{\mu_{d}}{\mu_{c}}$$

where $\mu_{c}$ is the dynamic viscosity of the continuous phase. A more generalized form of Oh that incorporates both droplet and continuous fluid viscosities can be expressed as:

(5) $$Oh=\frac{\mu_{c}}{\sqrt{\rho_{d}\;\sigma_{dc}\;d_{d}}}\lambda$$

In turbulent flows, droplet deformation and breakup are influenced by the turbulent energy dissipation rate (ε), which may be expressed in terms of relative velocity $U_{dc}$ as [11,36]:

(6) $$U_{dc}\;{\left(\varepsilon d_{d}\right)}^{1/3}$$

Using Equations (2), (6), We may be written in terms of ε as:

(7) $$We=\frac{\rho_{d}\varepsilon^{2/3}{d_{d}}^{5/3}}{\sigma_{dc}}$$

The maximum stable droplet size $\left(d_{max}\right)$ may be expressed in terms of ${We}_{crit}$ as:

(8) $$d_{max}={\left(\frac{{We}_{cr}\;\sigma_{dc}}{\rho_{d}\;\varepsilon^{2/3}}\right)}^{3/5}$$

Once the physical properties of the system ($\sigma_{dc}$ and $\rho_{d}$), energy dissipation rate (ε) and the value of ${We}_{crit}$ are known, $d_{max}$ can be calculated using Equation (8), and thus it provides a framework to characterize potential droplet breakage for a given initial size of the droplet. Several studies on droplet breakage have reported values of ${We}_{crit}$ for different systems. For fully turbulent flows and smaller viscosity ratios (λ<3), ${We}_{crit}$ may be assumed to be unity [6,37]. At larger values of λ, the value of ${We}_{cr}$ was found to increase with the value of λ [38] as:

(9) $${We}_{crit}=a\left(1+b\lambda^{c}\right)$$

Where a, b, and c are fitting constants that depend on the flow type (e.g., shear, elongational, or turbulent). For turbulent flow conditions, typical values are a∼0.5-1, b∼1 and c∼0.4-0.6.

This expression is particularly useful because it accounts for the effect of internal viscous resistance on the droplet’s deformability. As λ increases, the droplet becomes more resistant to internal circulation and deformation, requiring a greater inertial force (i.e., higher We) to overcome surface tension and cause breakup. Therefore, incorporating ${We}_{crit}\;\left(\lambda \right)$ into predictive models allows for more accurate determination of the breakup threshold across a wide range of droplet viscosities and flow conditions.

While Equation (8) is useful for estimating droplet breakage, the application of this is largely restricted to devices where energy dissipation rates are relatively small ($\varepsilon <10^{4}$ m^2^/s^3^). The energy dissipation rates realised by collapsing cavities generated by ultrasonic or hydrodynamic cavitation are orders of magnitude higher ($\varepsilon >10^{7}$ m^2^/s^3^). These extreme environments can lead to droplet deformation or breakup under different mechanisms than those underlying the formulation of Equation (8). For example, our previous study showed that interaction between a single cavity and a droplet was insufficient to cause breakup [10] despite generating very high energy dissipation rates ($\varepsilon \;∼10^{7}$ m^2^/s^3^). The likely reason is due to the very fast dynamics of cavity collapse. To further investigate droplet deformation and breakup under such rapid transient loading, in this work, we perform a numerical investigation involving two collapsing cavities adjacent to an oil droplet. This setup mimics multiphase interactions relevant to hydrodynamic cavitation-assisted emulsification, and allows us to assess whether the combined effect of two cavity collapses can induce oil droplet breakup. The details of the computational model are presented in Section 3. The results of numerical simulations are discussed in Section 4.

## 3 Computational model

In our previous work [10], we analysed cavity dynamics near a deformable oil droplet suspended in water and observed that the cavity jet was directed away from the droplet, causing deformation but not breakup. This finding motivated the present study, which aims to examine whether the interaction with two symmetrically positioned cavities can generate sufficient stress to induce droplet fragmentation. Based on the findings of Pandey et al. [10], we adopt a stand-off distance (γ) of 1.4 and β of 2.5, as these parameters were shown to yield maximum cavity jet velocity. The γ=1.4 is retained for the present investigation into the effects of σ, λ, and driving pressure (ΔP). To further explore the role of droplet size, the diameter is varied to assess the influence of β on deformation and breakup behaviour. The simulation parameters, along with the corresponding dimensionless parameters:We and Oh are summarised in Table 1.

**Table 1:** We and Oh for the range of σ, λ, β and ΔP

$d_{d}\;\left(10^{-6}\right)$**, m**	$d_{c}\;\left(10^{-6}\right)$**, m**	β**, −**	$\sigma_{dc}$**, N/m**	λ**, −**	$\Delta P(10^{7})$**, Pa**	${We}_{dc}\;\left(10^{3}\right)$**, −**	$Oh_{dc}$**, −**
50	20	2.5	0.01	5	1	49.5	0.224
			0.02			24.8	0.158
			0.03			16.5	0.129
			0.04			12.4	0.112
			0.05			9.9	0.100
1.5		0.075	0.03			0.49	0.746
12.5		0.63				4.13	0.258
25		1.25				8.25	0.183
50		2.5				16.5	0.129
100		5				33	0.091
50		2.5		1		16.5	0.026
				10			0.258
				100			2.585
				300			7.754
				500			12.923
			0.03	5	0.05	0.067	0.129
					0.1	1.5	
					0.3	4.83	
					0.5	8.17	
					1	16.5	
					5	83.2	
					10	167	
					50	833	

**Fig. 1:**
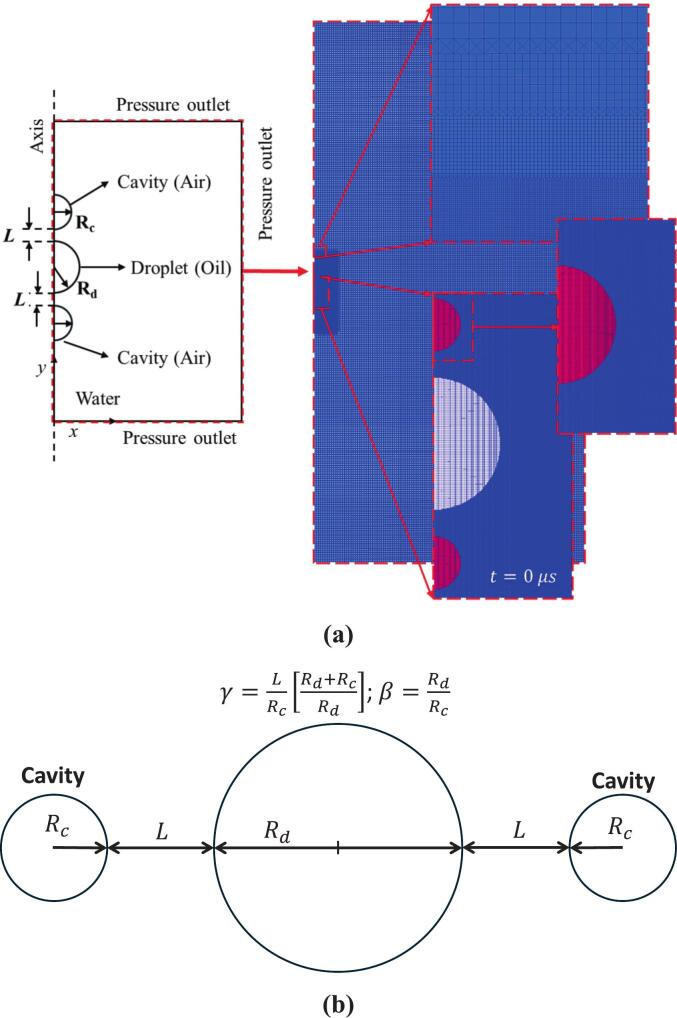
(a) Computational domain and grid distribution; (b) Stand-off parameter (γ) and size ratio (β) used in the present study.

To study these dynamics, we simulate the interaction of two air cavities with a centrally located oil droplet suspended in a continuous water medium, as illustrated in Fig. 1a. This configuration introduces axisymmetric, transient flow conditions that differ significantly from those observed under steady shear or homogeneous turbulence. The alignment of the cavities on opposite sides of the droplet creates a directional, focused force field, mimicking realistic scenarios in hydrodynamic cavitation-based fluidic devices such as vortex diodes, venturi, and orifices used during emulsification. A 3D axisymmetric computational domain is employed to reduce computational cost while retaining the essential physics of radial cavity collapse, which would otherwise necessitate high-cost full 3D modelling.

The conventional model of cavity dynamics typically emphasises inertial forces while neglecting the effects of mass and heat diffusion across the cavity wall, as well as phase change phenomena [32,39]. This model is relevant for cavities in water at normal ambient temperatures, where cavity dynamics are primarily governed by inertial and compressibility effects, while heat diffusion is considered negligible. Experimental evidence from Vogel et al. [40] supports this approach, demonstrating that over 80% of the energy released during a strong cavity collapse is emitted as a shock wave. Furthermore, gas diffusion through the cavity wall is negligible due to the significantly slower time scale ($\;∼10^{-1}-10^{0}\;$ s) of gas diffusion compared to the rapid oscillations of the cavity ($\;∼10^{-6}$ s). As a result, thermodynamic effects do not substantially influence the cavity's overall dynamics. The behaviour of a cavity filled with non-condensable gas (NCG) [41] differs from that of a cavity filled with water vapor, primarily due to phase change effects. For cavities containing water vapor, condensation leads to a reduction in cavity pressure and size over time [33]. To account for this, a one-time correction to the cavity pressure is implemented, reducing the pressure by a factor of 0.35 once the cavity reaches its maximum size during the first oscillation cycle [33]. A similar approach was employed by Fan et al. [42], who reduced the cavity's radius by a factor when it reached its maximum size to model the partial condensation of the cavity contents and align with experimental observations in subsequent oscillations. This correction enhances the accuracy of the model compared to the assumption of a non-condensable gas. The simplification provided by assuming NCG enables a more straightforward model. Additionally, the analysis assumes isothermal conditions, as heat conduction [39,40] has been found to exert minimal influence on the rapid dynamics of cavity oscillations. The influence of liquid viscosity and interfacial tension is incorporated into the model, as these factors play a significant role in the cavity dynamics and the interaction between the cavity and droplets [39].

To fully characterize the multiphase system, three σ are considered: the interfacial tension between the droplet and the continuous medium $\left(\sigma_{dc}\right)$, between the cavity and the continuous medium $\left(\sigma_{cav-c}\right)$, and between the cavity and the droplet $\left(\sigma_{cav-d}\right)$. These interfacial tensions govern the capillary forces acting at each interface and play a crucial role in determining the extent of droplet deformation and breakup under dynamic flow conditions. Additionally, three λ are defined to capture the shear and momentum transfer effects across the interfaces. These include the λ between the droplet and continuous medium $\left(\lambda_{d-c}\;\right)$, between the cavity and continuous medium $\left(\lambda_{cav-c}\;\right)$, and between the cavity and droplet $\left(\lambda_{cav-d}\;\right)$. In the present work, $\sigma_{dc}$​ is of primary interest, as droplet breakup is driven by the forces transmitted through the continuous phase during cavity collapse. The energy released from the collapsing cavity primarily propagates through the surrounding medium, making the droplet–continuous interface the most relevant to capture the deformation behaviour. Similarly, $\lambda_{d-c}$​, referred to as λ throughout the study, is used to characterize the droplet's internal resistance to deformation relative to the continuous phase. This choice aligns with the focus on how viscous damping and surface tension influence the fragmentation threshold under cavity-induced flow fields.

The physical phenomena governing the interaction between the cavity and the droplet exhibit symmetry about the central axis, prompting an axisymmetric approach for the present study. All fluids are treated as Newtonian. The continuous phase (water) and the oil droplet are both modelled as compressible, with the density of each phase described using Tait’s equation of state [39,43,44]. The oscillations of the cavity are triggered by imposing an elevated pressure, substantially higher than the atmospheric pressure conditions of the other two phases. The subsequent section outlines the model equations, boundary conditions, and solution strategies employed in the simulations.

### 3.1 Governing equations

A set of governing equations, encompassing mass, momentum, and energy conservation, is solved to compute the flow fields in the present study. These equations are as follows:

(10) $$\frac{\partial \rho }{\partial t}+\nabla \cdot \left(\rho u\right)=S_{c}$$

(11) $$\frac{\partial \left(\rho u\right)}{\partial t}+\nabla \cdot \left(\rho uu\right)=\;-\nabla p+\nabla ∙\left(\mu \nabla u\right)+\rho g+F_{s}+S_{m}$$

(12) $$\frac{\partial \left(\rho C_{p}T\right)}{\partial t}+\nabla \cdot \left(\rho C_{p}uT\right)=\;\nabla ∙\left(k\nabla T\right)-p\nabla ∙u+S_{e}$$

where u, p, T, ρ, μ, $C_{p}$, k, g, $F_{s}$, $S_{c}$, $S_{m}$, and $S_{e}$ are velocity, pressure, temperature, mixture density, mixture viscosity, mixture specific heat, mixture conductivity, gravity of acceleration, surface tension force, and source terms for mass, momentum, and energy, respectively.

To model the presence of multiple phases, a multi-fluid algebraic VOF approach is utilized. This method effectively captures interfaces between different phase pairs, rigorously conserving mass and handling complex interfaces [45]. The VOF technique has been successfully employed in prior research by Raman et al. [33] and Pandey et al. [10] to investigate cavity-droplet interactions and is adopted here to analyze the cavity dynamics near the oil droplet. The decision to employ the FVM with VOF over alternatives such as the Boundary Integral Method (BIM) or the Lattice Boltzmann Method (LBM) is based on its ability to capture sharp interfaces, manage large deformations, and accommodate topology changes [40,45,46]. Additionally, FVM with VOF seamlessly integrates the effects of viscosity and surface tension forces, which are critical for accurately modelling the interactions between the cavity and droplets [45,46].

In the VOF method, scalar transport equations [Equation (13)] are solved using a multidimensional universal limiter with explicit solution (MULES) to compute the volume fraction distribution [Equation (14)] for each phase in each computational cell [39,47].

(13) $$\frac{\partial \alpha_{i}}{\partial t}+\nabla \cdot \left(u\alpha_{i}\right)=0,\;\;i=1,2$$

(14) $$\alpha =\frac{V_{i}}{V}$$

where $\alpha_{i}$ represents the volume fraction of phase i, and $V_{i}$ and V are the volume of phase i and the total cell volume, respectively. The sum of the volume fractions of all the phases in a cell is always equal to one [Equation (15)].

(15) $$\sum \alpha_{i}=1$$

The mixture properties, such as density, viscosity, heat capacity, and conductivity, are determined using Equation (16).

(16) $$b\;=\sum \alpha_{i}b_{i}\;,\;\;\;\;i=1,2,3,\;\;\;b\in \left(\rho ,\;\mu ,\;C_{p},\;k\right)$$

To model the surface tension forces at the phase interfaces, a source term ($F_{s}$) is included in the momentum equation [Equation (11)]. This term is based on the continuum surface force (CSF) model, as described by Brackbill et al. [48]. The surface tension force ($F_{s}$) is given by:

(17) $$F_{s}=\sum_{i}\sum_{j\neq i}\sigma_{ij}\kappa_{ij}\delta_{ij}$$

where, $\sigma_{ij}$, $\kappa_{ij}$, and $\delta_{ij}$ are surface tension coefficient, curvature, and Dirac delta function, respectively. Subscripts i, j denote different phase pairs. The curvature ($\kappa_{ij}$) is computed as:

(18) $$\kappa_{ij}=-\nabla ∙\left(\frac{\alpha_{j}\nabla \alpha_{i}-\alpha_{i}\nabla \alpha_{j}}{\left|\alpha_{j}\nabla \alpha_{i}-\alpha_{i}\nabla \alpha_{j}\right|}\right)$$

and the $\delta_{ij}$ is given as:

(19) $$\delta_{ij}=\alpha_{j}\nabla \alpha_{i}-\alpha_{i}\nabla \alpha_{j}$$

The $\delta_{ij}$ ensures that the surface tension force only acts at the interface between different phases.

To model the compressibility of the liquid and gaseous phases, Tait’s equation of state (EoS) [Equation (20)] [43,49] is used. This equation accounts for fluid density variations over a wide range of pressures (from 0.1 MPa to 50 MPa), which is important during cavity collapse (high pressure) and growth (low pressure). The EoS also models the nonlinear compressibility of liquid and vapor phases, providing a direct relationship with the isentropic bulk modulus [50]. The equation is given as:

(20) $$p=\left(p_{0}+B\right){\left(\frac{\rho }{\rho_{0}}\right)}^{N}-B$$

Here, $p_{0}$, $\rho_{0}$, N, and B are reference pressure, reference density, adiabatic exponent and empirical parameter, respectively. For B=0, this equation reduces to the ideal gas law. Properties for the fluids are taken from the study by Raman et al. [33] and are summarized in Table 2. The reference pressure (10320 Pa) and density (0.12 kg/m3) for air are selected so that Tait’s equation closely matches the behaviour of an ideal gas undergoing adiabatic compression or expansion. At this specific reference state, the Tait equation becomes equivalent to the adiabatic ideal gas law, ensuring accurate modelling of compressible air behaviour. An adiabatic approximation is used to model the gas pressure within the cavity, as the cavity dynamics are primarily driven by inertia and compressibility effects, with heat diffusion having negligible influence [40].

**Table 2:** Parameters and their values utilized in Equation (20) [33].

**Parameter**	**Air**	**Water**	**Oil**
Reference density, ($\rho_{0},$ kg/m^3^)	0.12	998.2	960
Reference pressure ($p_{0}$, Pa)	10,320	101,325	101,325
Adiabatic exponent (N)	1.33	7.15	6.4
Empirical parameter (B, MPa)	0	404.6	150

### 3.2 Computational domain and boundary conditions

As previously mentioned, an axisymmetric computational domain is employed in this study. The domain represents a portion of a large cylindrical space filled with a continuous phase (water), where two cavities (air) of identical size and pressure difference, along with a droplet (oil) of the same size, are positioned close to each other (Fig. 1a). The size of the computational domain is chosen to be 100 times the maximum radius of the cavities to minimize the influence of boundary conditions on the interaction between the cavities and droplets. Pressure outlet and wave-transmissive boundary conditions are applied at the outlets to simulate a larger fluid domain. Initially, the entire domain is at atmospheric pressure, except for the cavities.

The size ratio (β) and stand-off parameter (γ) are defined as:

(21) $$\beta =\frac{d_{d}}{d_{c}}$$

(22) $$\gamma =\varphi \left(1+\frac{1}{\beta }\right)$$

Where, $d_{c}$ is the initial radius of the cavity (fixed at 20 μm), $d_{d}$ is the initial radius of the oil droplet, $\varphi =L/d_{c}$, L is the distance between the initial cavity surface to the oil droplet surface as shown in Fig. 1b. The definition of γ in the present work is different from that of Orthaber et. al [44], and the relationship between γ of the present work and Orthaber et. al [44] is also provided in our previous work [10].

### 3.3 Numerical solution and grid independence

Numerical simulations are conducted using OpenFOAM, an open-source computational fluid dynamics software based on the FVM. The present study employs the compressibleMultiphaseInterFoam solver, which has been widely adopted in previous investigations addressing cavity-droplet interaction phenomena [10,33,34]. The governing equations are discretised using the FVM framework and solved through the PIMPLE algorithm. The procedure begins with the calculation of phase densities using Equation (20). Subsequently, the volume fraction transport equations [Equations (13,14)] are solved, from the mixture properties [Equation (16)] and surface tension forces [Equations (17-19)] are derived. These properties are then incorporated into the momentum and continuity equations [Equations (10-12)] to resolve the flow field. This iterative process continues until the prescribed final simulation time is reached. An adaptive time-stepping approach was employed, regulated by a maximum Courant number of 0.05, to ensure numerical stability and accuracy. The effect of gravity is neglected as it does not play any role in the cavity dynamics, especially on the cavity jet formation (as shown in Section S2 of the supplementary information). Detailed setup instructions for the simulation, including case configuration and post-processing procedures in OpenFOAM, are provided in the Supplementary Information of our previous publication [10].

A non-uniform structured Cartesian grid having an aspect ratio of one was used to discretize the simulation domain. It offers a consistent framework for interface capturing, reducing numerical diffusion, and ensuring a precise and sharp interface representation. To enhance the computational efficiency, only the grids around the cavity where the maximum deformation occurs are refined (Fig. 1a). Three different grid sizes were considered, that is, 0.25 µm, 0.31 µm and 0.4 µm. The influence of grid size on maximum velocity and radius is shown in Table 3. Moreover, the Richardson extrapolation method [51] (Table 3) is used with the maximum velocity obtained at t=13 μs as its parameter. The $U_{max}$ is an appropriate parameter to test the influence of the grid since cavity jet velocity is responsible for the droplet deformation and fragmentation. The cavity jet velocity gradient was also used in the evaluation of the energy dissipation rate (ε).

**Table 3:** Minimum grid size and corresponding maximum velocity.

**Refinement**	**Refinement Ratio (**r**)**	**Grid size, μm**	**Maximum velocity**	**m/s**
h1	1	0.25	V1	139
h2	$r_{21}=\;h_{2}/h_{1}=1.24$	0.31	V2	141
h3	$r_{32}=\;h_{3}/h_{2}=1.29$	0.40	V3	118

Using the Richardson Extrapolation formula [51], the value of velocity at mesh size approaches zero ($V_{h\to 0}$) was estimated as:

(23) $$V_{h\to 0}=\;V1+\left(\frac{V1-V2}{{r_{21}}^{p}-1}\right)\;\;\;135.28\;m/s$$

Where V1, V2 are the maximum velocities of the cavity jet obtained on grid 1 and grid 2, $r_{21}$ is the grid refinement ratio of grid 2 to grid 1. These calculations led to an estimated discretization error of 4%, confirming that further mesh refinement would not significantly alter the results. Based on this analysis, the grid with a minimum cell size of 0.31 µm (h2) was selected for all subsequent simulations. It offers a satisfactory balance between accuracy and computational cost, while maintaining fidelity in capturing key flow dynamics such as jet formation and energy dissipation near the cavity interface.

## 4 Results and discussions

This section presents a detailed analysis of droplet deformation and breakup induced by the collapse of two cavities using the VOF. The DNS simulations are carried out in an axisymmetric cylindrical domain, with particular attention to interfacial dynamics, pressure distribution, and velocity fields during cavity collapse. Although the simulation results visually appear as 2D slices, the underlying computations were performed in a 3D wedge-shaped axisymmetric domain to accurately capture the full physical behaviour while maintaining computational efficiency.

### 4.1 Verification and validation

**Fig. 2:**
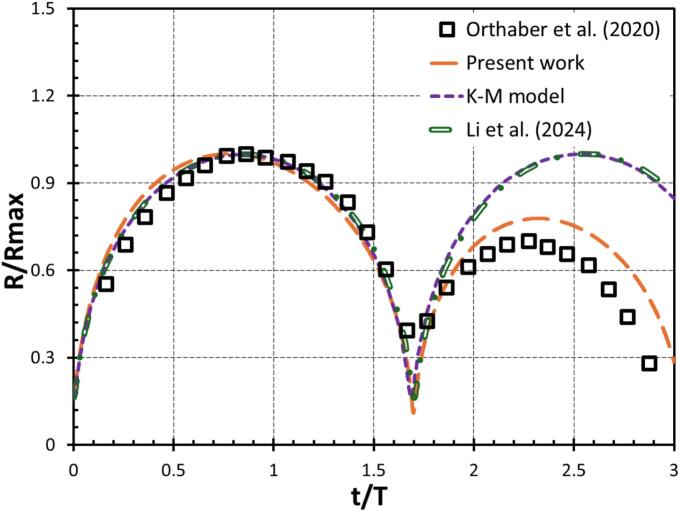
Comparison of the present numerical study with the existing published work.

The present simulation methodology is first validated by comparing the simulation results with previously published studies on cavity dynamics. Validation was conducted for two distinct scenarios to ensure robustness. Initially, the temporal evolution of a cavity within a liquid pool was compared with the experimental data reported by Orthaber et al. [44]. In addition, for quantitative validation, we employed the modified Rayleigh–Plesset (RP) model developed by Li et al. [52], which incorporates surface tension and viscosity and was shown to be consistent with experimental data. Applying the same model to our simulation case, we found good agreement in capturing key physical features, as shown in Fig. 2. It can be seen that the results of the present work agree well with all three reported results for the first cycle. For the second cycle, the K-M model and Li et al. [52] do not consider the phase change. Therefore, their results deviate from the experimental data, while the results obtained in the present work are closer to the experimental data reported by Orthober et al. [44].

**Fig. 3:**
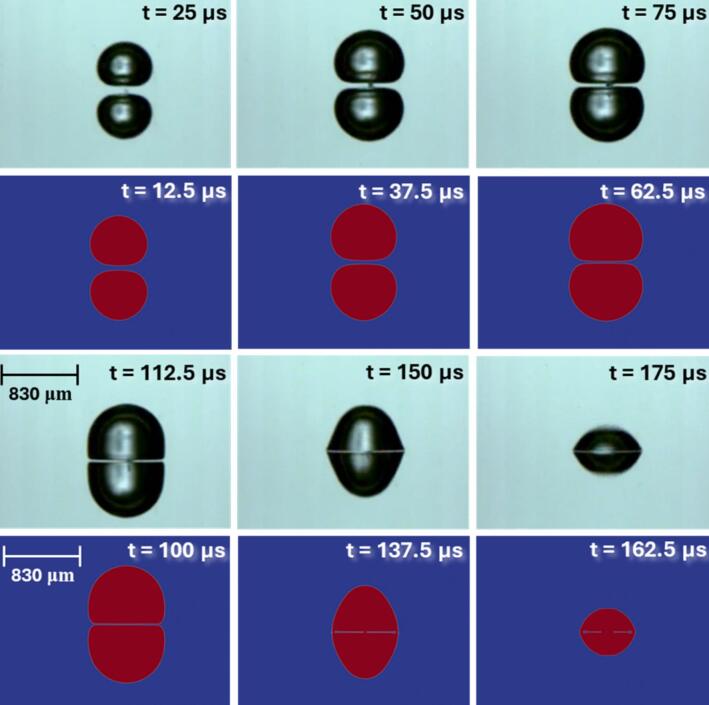
Interactions of two cavitation bubbles generated in water (Top: Luo and Niu [53]; Bottom: Present solver validation).

In the second case, the numerical model is qualitatively validated against the experimental observations reported by Luo and Niu [53]. This particular study was selected for comparison because the current work also involves the interaction of two cavities with a droplet. Luo and Niu [53] did not report the initial cavity radius in their study. They only reported that the initial separation between the two cavities at inception was 1.23mm, and the maximum cavity size was reported as 830μm at 112.5μs. We formulated our simulation case using this reported information. The initial radius of the cavity was estimated using the relation $R_{initial}=R_{max}\;/6.25$, as proposed by Orthaber et al. [44]. Since the initial pressure inside the cavities was not specified in the experimental study, we adopted an approach consistent with our previous work [10], where the initial pressure was varied to approximate the conditions of laser-induced cavitation. Based on that methodology, the initial cavity pressure in the current simulations was taken as $10^{7}\;Pa$. Because of the uncertainties involved in estimating the initial conditions for our simulations, there may be some differences in the simulated and experimental results. The details of these additional simulations (geometry, mesh and other details) are included in Section S1 of the supplementary information. The comparison of the simulated results with the data reported in [53] is shown in Fig. 3. The comparison of simulated and experimental results indicates that there is a difference of about 12.5μs. This difference may arise due to differences in the initial condition and inception time of cavitation in the two cases. Ignoring this difference, it can be seen from Fig. 3 that the two cavities expand synchronously to the maximum volume, and during the expansion, the adjacent side surfaces gradually become planar, and the liquid in the middle of the two cavities is gradually squeezed by the expansion of them. When expanded to the maximum volume, the middle layer becomes a very thin liquid film. Despite the thinning, the two cavities did not fuse, as can be seen from Fig. 3. During the shrinking process, the adjacent surfaces of the bubbles still appear planar, while the surfaces away from each other violently shrink toward the centre of the respective cavities. There was no obvious fusion between the two cavities during the shrinking phase. The simulated results captured these experimental observations well (refer to Fig. 3).

### 4.2 Drop breakup

**Fig. 4:**
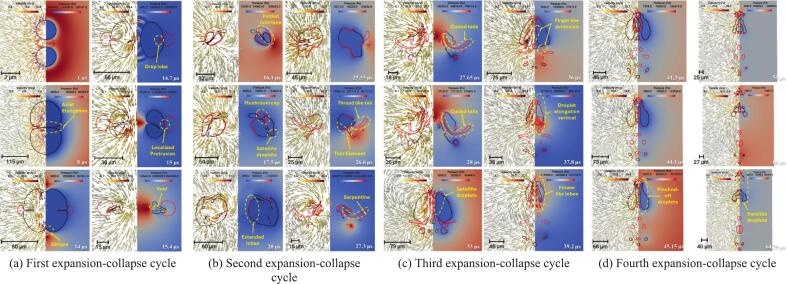
Velocity contour with pressure magnitude on vector showing drop breakage via two collapsing cavities for $\sigma_{dc}=0.01$ N/m, $\mu_{d}=0.005$ Pa.s at β=2.5 (black colour line is the cavity, and the red colour line is the droplet). (For interpretation of the references to color in this figure legend, the reader is referred to the web version of this article.)

Simulated time-resolved sequence of the interaction between two cavities and a centrally positioned oil droplet suspended in water (continuous phase) is shown in Fig. 4. The droplet (outlined in red) is located symmetrically between the two air cavities (black lines). Each frame shows the flow dynamics in a split format: the left half illustrates velocity vectors representing fluid motion, while the right half displays pressure contours capturing local pressure gradients. These frames capture the transient evolution of the droplet-cavity system at discrete time points, revealing key physical mechanisms governing droplet deformation and potential breakup.

At 1 µs, the cavities experience rapid expansion due to high internal pressure (∼10^7^ Pa), generating a radially outward flow in the surrounding water. This flow creates diverging velocity vectors and initiates interfacial stresses on the adjacent oil droplet. Initially spherical, the droplet begins to elongate axially under the influence of tensile forces along the cavity axis. The central region undergoes pressure reduction, which contributes to stretching the droplet. By 8 µs, as shown in Fig. 4a, the cavities approach their maximum size, sustaining a jet-like flow that continues to pull the surrounding fluid outward. These dynamics impose symmetric tensile forces that extend the droplet further, with high-shear zones developing near the interface due to velocity gradients. The collapse of the cavities begins at 14 µs, reversing the flow direction and initiating compression toward the cavity centres. This inward movement leads to lateral compression of the droplet, forming indentations along its sides. As collapse intensifies at 14.7 µs, strong inward velocity fields exert normal stresses on the droplet, driving oil from the centre toward the edges and forming pinch-off lobes. In Fig. 4a, signs of vorticity and shear-induced interface curling are apparent, suggesting the early formation of vortex rings. By 15.4 µs, one of the cavity nears complete collapse, producing a transient void that triggers a fluid influx and intensifies shear layers around the droplet. The interface becomes highly deformed and asymmetric, with lobes showing high curvature, indicating possible topological transitions such as pinch-off or entrainment. The second expansion and collapse cycle begins then.

Interfacial folding becomes prominent by 16.1 µs, as evident in Fig. 4b, caused by asymmetric cavity collapse and associated non-uniform pressure gradients. The surrounding fluid converges inward with a rotational component, folding the droplet interface and triggering interfacial buckling. At 17.5 µs, localized vortex formation and Rayleigh–Taylor-like instabilities create a mushroom cap structure at the droplet front. Pinch-off of small satellite droplets occurs due to localized necking and thinning, a sign of capillary-driven breakup. Sustained tangential flow along the droplet interface by 20 µs promotes asymmetric stretching and amplifies lobe formation through vortex ring activity. At 25.55 µs, flow reorganisation causes opposing shear fields around the droplet, creating a twisted, stretched core. This is followed by the emergence of thin filaments and a thread-like tail at 26.6 µs, both classic results of viscous elongation under velocity gradients. These features, visible in Fig. 4b, suggest that shear overcomes interfacial tension momentarily, causing the droplet to split into separate regions. A serpentine morphology forms at 27.3 µs as intense vorticity wraps the interface, bending the droplet back on itself. Spiral vector patterns indicate strong rotational flow and a helical droplet trajectory, typical in turbulent, vortex-rich environments. The third expansion cycle starts here.

By 27.65 µs, Fig. 4c shows curled tails forming due to asymmetric vortex shedding around the droplet. Localized spiraling motions suggest Kelvin–Helmholtz-type instabilities arising from sharp velocity discontinuities at the oil–water interface. Continued rotation at 28 µs further stretches and folds the tails, while pressure gradients drive upper-section elongation and lower-section compression, enhancing asymmetry. At 33 µs, tip streaming and filament rupture lead to the release of satellite droplets, driven by flow-induced extension. A finger-like extension, characteristic of Saffman–Taylor instability, forms at 36 µs as lower-viscosity water pushes the oil outward. Vertical elongation dominates by 37.8 µs, and a narrow shear band develops, aligning with a high-tension axis that pulls oil upward. Flower-like lobes emerge by 39.2 µs, generated by competing flow directions that produce multi-point curvature variations along the interface. Substantial deformation is seen at 42.3 µs, where Fig. 4d captures a droplet shaped by prior cavity collapse into a highly lobed, asymmetric structure with thin necks. A residual pressure gradient persists, compressing the interface unevenly. Vortex rings and velocity shear become more defined at 44.1 µs, especially near the upper tip. These circulatory flows thin the necks and accelerate interfacial breakup. At 45.15 µs, several pinch-off events result in small daughter droplets, surrounded by strong local flow fields. These droplets rotate and translate under the influence of ambient fluid dynamics, while the remaining main droplet loses mass and remains elongated. The fourth expansion cycle starts.

By 50 µs, asymmetric velocity fields continue to stretch the main droplet and move the satellite droplets, as shown in Fig. 4d. Though cavity collapse has ended, its legacy remains in pressure and shear distributions that prolong fragmentation. At 60 µs, the system enters a stage dominated by fragment advection and interfacial relaxation. Detached droplets move with local flow fields and interact with surrounding eddies. The remaining portions of the main droplet exhibit undulating interfaces, driven by trapped shear layers and residual vorticity. These patterns suggest ongoing emulsification processes. At 64.75 µs, the system approaches a late relaxation phase, with satellite droplets clearly separated and migrating downstream. The flow field stabilizes, showing mild gradients, but localized vortices remain near the original droplet location. These may influence further droplet evolution through secondary pinch-off or delayed coalescence. At this stage, interfacial tension and viscous dissipation dominate, working to minimize surface energy and redistribute the droplet mass across the surrounding fluid. Influence of various parameters is discussed in the following sections.

### 4.3 Effect of interfacial tension$\left(\sigma_{dc}\right)$

**Fig. 5:**
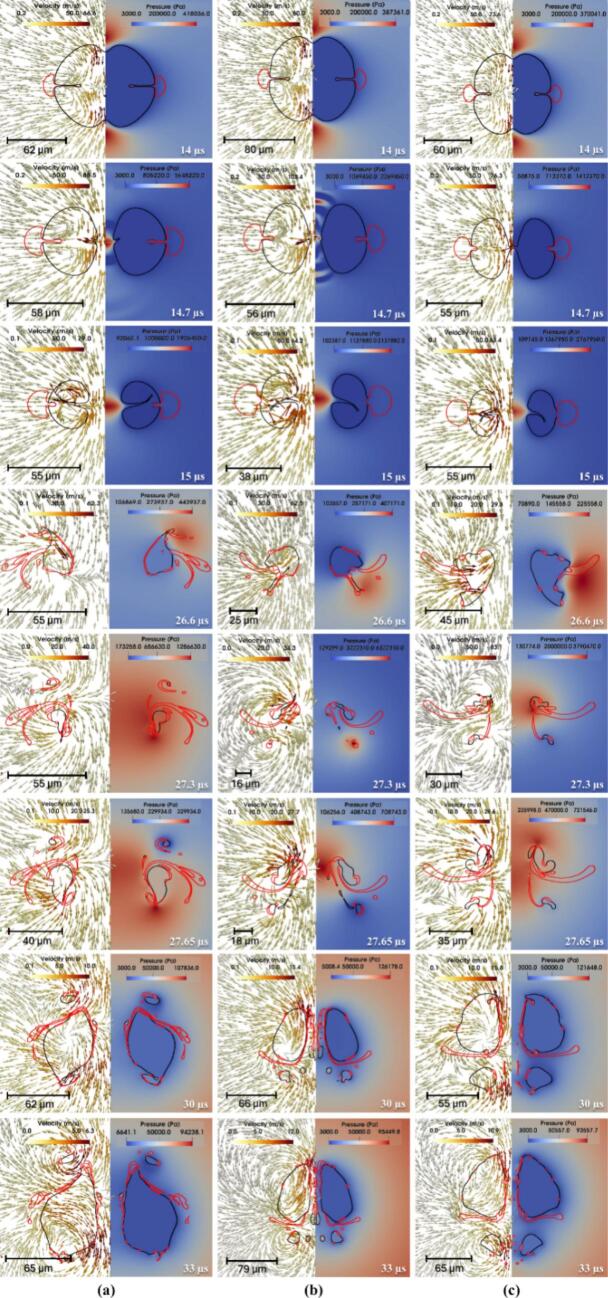
Velocity contour with pressure magnitude on vector showing the difference in drop breakage via two collapsing cavities (black color line is cavity and red color line is droplet) for different interfacial tension $\left(\sigma_{dc}\right)$ (a) 0.01 N/m; (b) 0.03 N/m and (c) 0.05 N/m at $\mu_{d}=0.005$ Pa.s,. (For interpretation of the references to color in this figure legend, the reader is referred to the web version of this article.)β=2.5

Influence of interfacial tension on the transient evolution of droplet deformation and breakup resulting from the collapse of two adjacent air cavities in water is shown in Fig. 5. These simulations are conducted at a fixed initial driving pressure $\Delta P=10^{7}$ Pa, with droplet viscosity $\left(\mu_{d}\right)\;$ of 0.005 Pa·s and β=2.5. It can be seen that at t=14 µs, cavity collapse begins, generating inward-directed velocity fields and steep pressure gradients around the interstitial zone. This results in significant compressive stress on the droplet, triggering its deformation. Between 14.7 to 15 µs, the cavities collapse rapidly, intensifying the surrounding pressure and producing a strong elongational flow. The droplet elongates axially, especially for the case with $\sigma_{dc}=\;0.01\;$ N/m, where interfacial tension is insufficient to counteract the imposed stresses. In contrast, for $\sigma_{dc}=0.05$ N/m, the droplet retains a more spheroidal shape due to higher resistance. The vertical asymmetry primarily arises from the dynamics of a pinch-off droplet formed during the initial expansion phase of the two cavities (refer to Section S3 of the supplementary information for more details). The detachment and subsequent motion of this droplet induce local asymmetry in the pressure and flow fields, which in turn lead to the formation of a directional jet and vertical displacement. The pinch-off of droplet is stochastic in nature and varies slightly among simulations. While this does introduce vertical asymmetry, it does not significantly alter the overall collapse dynamics or the key breakup mechanisms under investigation, as seen in Fig. 5.

At 26.6  µs, the asymmetric collapse of the surrounding cavity initiates a dynamic interplay of shear and extensional flow fields around the droplet. Velocity vectors reveal the emergence of complex vortical structures, while pressure contours show highly localized high- and low-pressure regions that contribute to significant distortion of the droplet interface. In the low interfacial tension case ($\sigma_{dc}=\;0.01$ N/m), the droplet elongates considerably, forming fine ligaments and secondary structures that indicate the onset of interfacial instability. As the collapse progresses to 27.3 µs, the breakup process intensifies. Growing instabilities, driven by strong inertial and pressure gradients, cause portions of the interface to detach. Droplets with higher interfacial tension exhibit comparatively limited fragmentation and show signs of morphological recovery due to the stabilizing influence of interfacial tension. By 30  µs, the droplet with the lowest interfacial tension ($\sigma_{dc}=\;0.01$ N/m) has fragmented into multiple ligaments and satellite droplets. The intermediate case ($\sigma_{dc}=\;0.03$ N/m) displays partial breakup and elongation, while the highest tension case ($\sigma_{dc}=\;0.05$ N/m) retains a largely intact, rounded form with minimal surface disruption. At 33  µs, the differences in outcome across interfacial tension values become even more pronounced. The final velocity and pressure fields, indicated by red contours, clearly demonstrate that lower interfacial tension promotes increased interfacial area generation via fragmentation, whereas higher tension suppresses breakup. These findings collectively illustrate the critical balance between inertial forces imposed by cavity collapse and the moderating influence of interfacial tension in governing droplet deformation and breakup pathways.

### 4.4 Effect of viscosity ratio(λ)

**Fig. 6:**
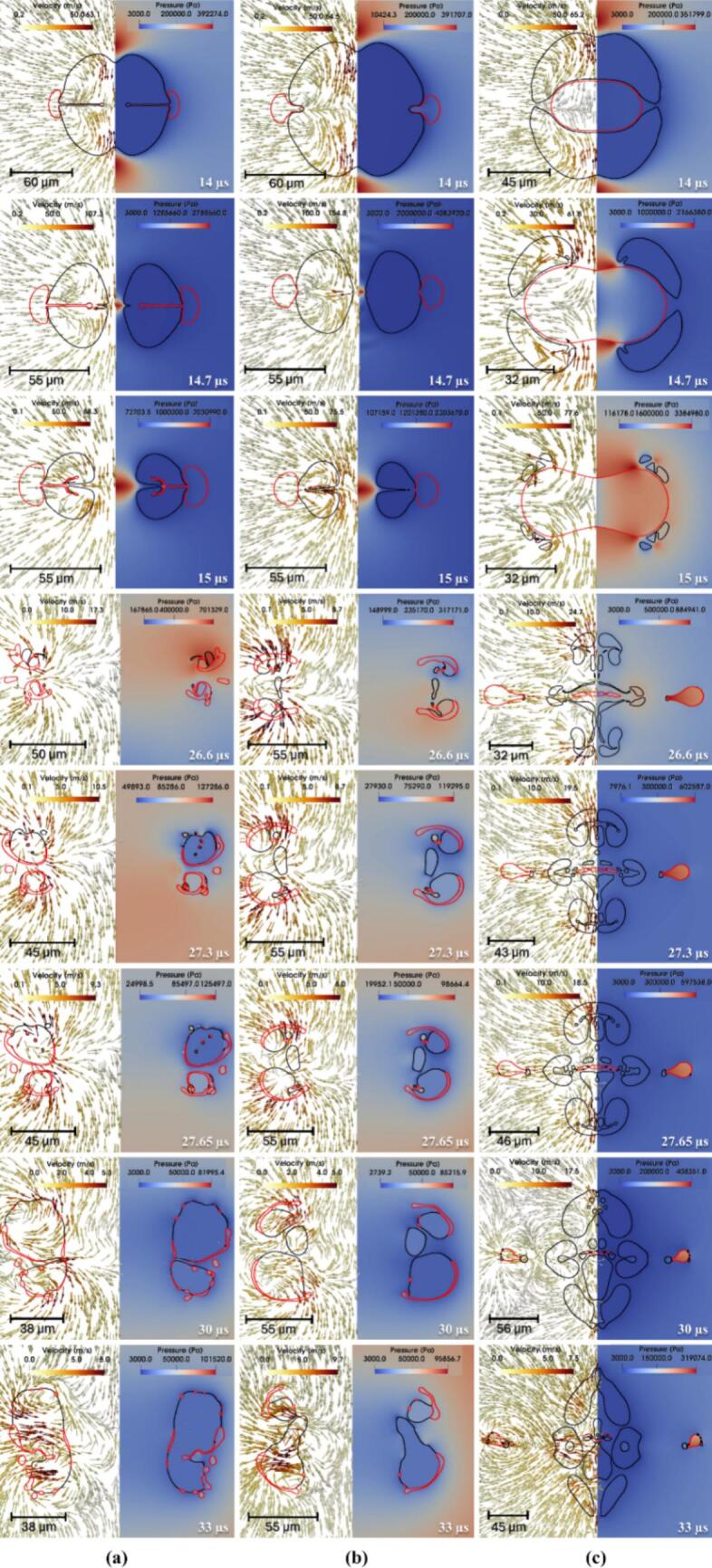
Velocity contour with pressure vector showing drop breakage via two collapsing cavities for different viscosity ratio (λ) (a) 1; (b) 100; (c) 500 at $\sigma_{dc}=0.03$ N/m and β=2.5

The breakup dynamics of an oil droplet subjected to two collapsing cavities are strongly influenced by the viscosity ratio (λ), as shown by simulations conducted at a constant $\Delta P\;=\;10^{7}$ Pa, $\sigma_{dc}=\;0.03$ N/m and β=2.5. The simulated results are shown in Fig. 6. The three cases are considered, λ=1,100 and 500, representing low, intermediate, and high viscosity ratios, respectively.

In the case of λ=1, the oil phase deforms rapidly due to the minimal resistance offered by its viscosity. At early times (14-15 µs), the cavity collapse produces nearly symmetrical pressure fields and converging velocity vectors that compress the droplet laterally. By 26.6 µs, vortical structures begin to wrap around the droplet, initiating necking and protrusions. The droplet’s low internal resistance allows these disturbances to grow swiftly. By 27.65 µs, the droplet is fragmented into multiple filaments and satellite droplets, which continue to evolve through inertial and interfacial forces. The final frames (30-33 µs) show a highly dispersed structure with complex flow interactions, demonstrating the high susceptibility of low-viscosity droplets to breakup under strong external flows.

In contrast, when λ=100, the droplet exhibits significantly higher viscosity relative to the continuous phase, which delays deformation.. Initially (14-15 µs), the cavity collapse appears similar to the λ=1 case, but the internal flow within the droplet is notably weaker, indicating that viscous stresses suppress internal circulation. As a result, deformation occurs more gradually. By 26.6-27.3 µs, the droplet shows signs of elongation and localized thinning, but full disintegration is not yet achieved. The pressure gradient still acts on the interface, generating ligaments, though their propagation is less extensive compared to the low-viscosity case. At 30 µs, partial fragmentation is observed, and by 33 µs, the droplet displays large residual segments, some with enclosed flow circulation, indicating that high viscosity inhibits rapid disintegration and tends to preserve droplet integrity.

For λ=500, the droplet behaves almost as a semi-rigid body. The cavity collapse (14-15 µs) generates similar external fields as in other cases, but the droplet deformation is minimal due to strong internal resistance to shear and elongation. Even by 26.6-27.3 µs, only moderate shape distortion is visible, and the internal pressure field remains relatively uniform. The cavities exert force on the droplet surface, but the viscous dissipation within the oil phase absorbs much of this energy. Ligament formation is largely suppressed, and instead, the droplet develops elongated but coherent structures. At 30-33 µs, the droplet remains intact, albeit with indented or flattened sides, signifying that extremely viscous droplets resist breakup even under symmetric, high-energy cavity collapse.

### 4.5 Effect of size ratio(β)

**Fig. 7:**
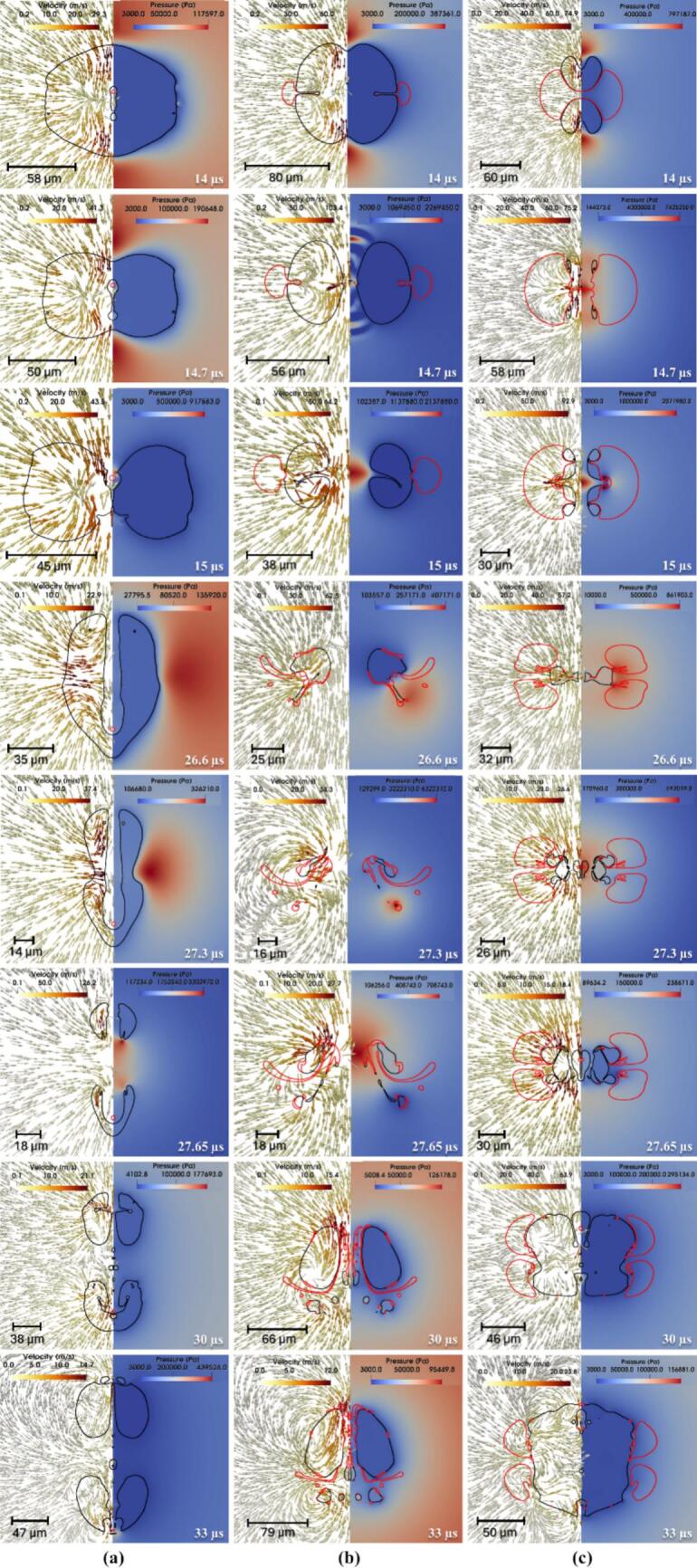
Velocity contour with pressure vector showing drop breakage via two collapsing cavities for different size ratios (β) (a) 0 .075; (b) 2.5; (c) 5 at $\sigma_{dc}=0.03$ N/m and $\mu_{d}=0.005$ Pa.s.

The dynamics of droplet breakup induced by the collapse of two cavities are strongly influenced by the β, as shown in Fig. 7. Simulations were performed at a constant driving pressure ($\Delta P\;=\;10^{7}$ Pa), $\sigma_{dc}=\;0.03$ N/m and $\mu_{d}=\;0.005$ Pa·s, examining three cases of β, which are 0.075,2.5 and 5.

For β=0.075, the droplet is much smaller than the collapsing cavities and is positioned symmetrically between them. In the early stages (14-15 µs), the cavities collapse symmetrically inward with minimal disturbance, generating moderate pressure fields that squeeze the droplet uniformly. Due to its small size and surface area, the droplet resists significant deformation and remains largely intact. The velocity field shows converging vectors, but no major circulation or vortices are generated. As the cavities fully collapse (27-30 µs), the droplet experiences only mild deformation, with no evident breakup, indicating that small droplets are less sensitive to pressure-induced fragmentation under symmetric collapse. In the intermediate case (β=2.5), the droplet size is comparable to the cavity diameter, leading to a much more pronounced interaction. Early stages again show symmetric collapse. By 15 µs, the pressure around the droplet increases significantly, and strong shear develops at the oil–water interface. As the cavities implode, their generated flow fields begin to wrap around the droplet, forming distinct vortical structures by 26.6 µs. These vortices stretch the droplet laterally and pull it apart, aided by the pressure gradients. By 27.65-30 µs, the droplet exhibits signs of rupture, forming ligaments and smaller sub-droplets. This scenario represents a balance where droplet inertia, interfacial tension, and cavity-induced flow all contribute strongly to deformation and eventual breakup. For β=5, where the droplet is significantly larger than the cavities, the interaction becomes highly asymmetric. From the onset (14 µs), the cavity collapse is distorted by the droplet's presence, particularly due to the obstruction it imposes. The pressure field around the cavities becomes non-uniform, and velocity vectors curve strongly around the droplet’s interface. At 15 µs, high-shear zones emerge, and by 26.6-27.3 µs, the droplet is engulfed by complex vortical motions. The collapsing cavities, now deflected sideways and downward, create strong rotational flows, which induce large-scale deformation in the droplet. Multiple ligaments and filamentary structures emerge by 30-33 µs, indicating advanced stages of breakup. The cavity energy is effectively transferred into the droplet via pressure waves and vortices, demonstrating that larger droplets experience extensive interfacial disruption and fragmentation under cavity-induced collapse.

In summary, the size ratio β plays a crucial role in governing the droplet-cavity interaction dynamics. Small droplets (β=0.075) experience minimal disruption and remain stable, while moderate-sized droplets (β=2.5) are optimally positioned for efficient energy transfer and breakup. Large droplets (β=5), in turn, significantly alter the symmetry of collapse and undergo intense deformation driven by complex, non-uniform pressure and shear fields.

### 4.6 Effect of driving pressure(ΔP)

**Fig. 8:**
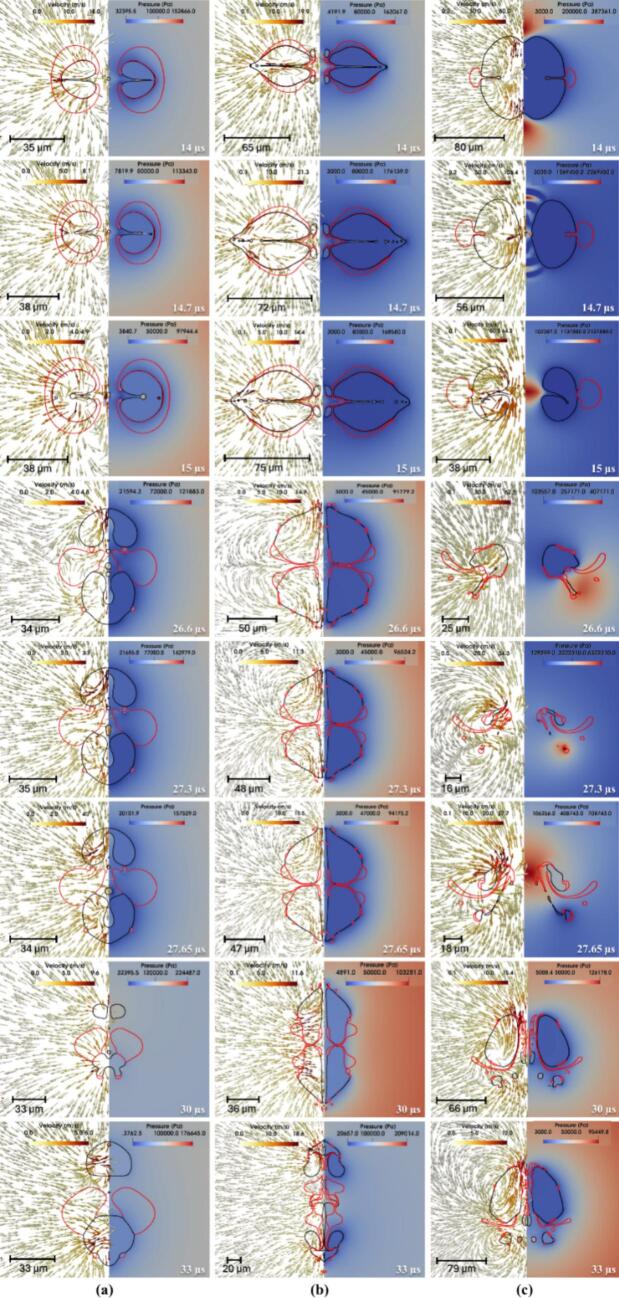
Velocity contour with pressure magnitude on velocity vector showing drop breakage via two collapsing cavities for different ΔP (a) ${1\;x\;10}^{6}$ Pa; (b) ${5\;x\;10}^{6}$ Pa and (c) ${1\;x\;10}^{7}$ Pa at $\sigma_{dc}=0.03$ N/m, $\mu_{d}=0.005$ Pa.s and β=2.5

The dynamics of droplet breakage induced by two collapsing cavities under varying ΔP (a) $1\times 10^{6}$ Pa, (b) $5\times 10^{6}$ Pa, and (c) $1\times 10^{7}$ Pa are investigated while maintaining a constant $\sigma_{dc}$ (=0.03N/m), $\mu_{d}=0.005$ Pa·s, and β=2.5 as shown in Fig. 8. The velocity contours combined with pressure vector fields provide insights into the evolution of cavity jets, pressure propagation, and energy transfer mechanisms driving droplet deformation and fragmentation. At 14 µs, all three cases exhibit the early stages of cavity collapse, where the jets formed due to cavity implosion begin to travel toward the denser water medium. In the $1\times 10^{6}$ Pa case, the jet is weak, showing minimal pressure gradients and velocity around the droplet, which remains almost spherical. For $5\times 10^{6}$ Pa, the cavity jets are stronger, inducing noticeable indentation on the droplet's sidewalls. The $1\times 10^{7}$ Pa case shows a much sharper and focused jet directed at the droplet, with increased velocity magnitude and pressure gradient, initiating significant droplet deformation at an early stage.

By 26.6 µs, the effects of cavity collapse become more distinct. At $1\times 10^{6}$ Pa, the deformation is mild, with the droplet only slightly compressed and pressure contours spreading gently around it. In contrast, for $5\times 10^{6}$ Pa, the droplet shows clear pinching in the midsection with stronger velocity fields and pressure differences acting along the axis of symmetry. The $1\times 10^{7}$ Pa condition results in a highly deformed droplet, with elongated shapes and early signs of fragmentation due to intense pressure and jet impact, indicating rapid energy transfer from collapsing cavities to the droplet. At 30 µs, the divergence in dynamics becomes more pronounced. For $1\times 10^{6}$ Pa, the droplet remains mostly intact with minor internal vortices and energy dissipation. In $5\times 10^{6}$ Pa, multiple internal structures start to form within the droplet, signalling early fragmentation. The pressure and velocity fields around the droplet are significantly elevated. The $1\times 10^{7}$ Pa scenario now features extensive deformation with clear filament-like structures and vorticity, showing signs of multiple pinch-off points and droplet fragmentation due to intense interaction with the surrounding fluid field. Finally, by 33 µs, the results establish the influence of initial cavity pressure on droplet breakup. In $1\times 10^{6}$ Pa, the droplet undergoes minor distortion, retaining most of its integrity. For $5\times 10^{6}$ Pa, the droplet is visibly fragmented into smaller masses due to strong cavity jet interaction and induced shear. The most extreme case, $1\times 10^{7}$ Pa, displays violent fragmentation with widespread formation of daughter droplets and vortex rings, highlighting maximum energy dissipation and breakup efficiency. The continuous escalation in cavity jet velocity and pressure with increased initial cavity pressure demonstrates that higher pressures accelerate deformation onset and promote more violent droplet disintegration over time.

In summary, as the initial cavity pressure increases, the cavity jet velocity, pressure gradients, and energy delivered to the droplet also increase, resulting in a shift from mild deformation to full fragmentation. This confirms the critical role of cavity pressure in governing droplet breakup dynamics in hydrodynamic cavitation-based systems.

### 4.7 Deformation and fragmentation

**Fig. 9:**
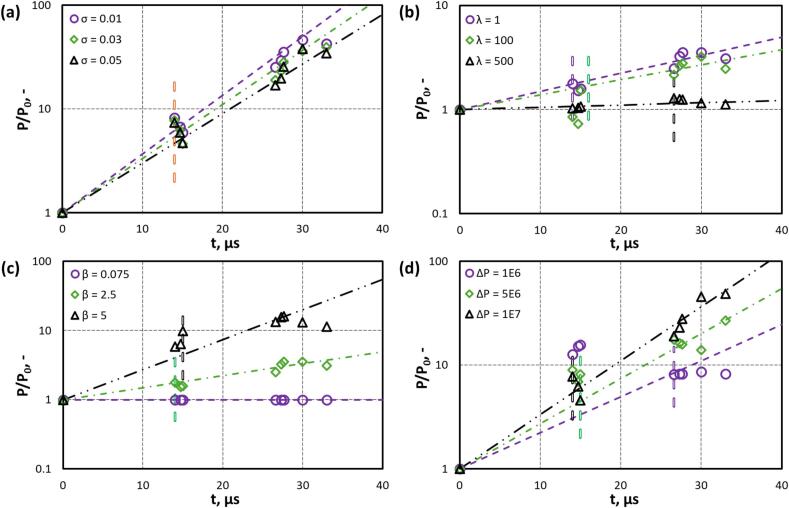
Variation of $P/P_{0}$ with time for different (a) σ at λ=5, $\Delta P=10^{7}$ Pa and β=2.5;(b) λ at $\sigma_{dc}=0.03$ N/m, $\Delta P=10^{7}$ Pa and β=2.5; (c) β at $\sigma_{dc}=0.03$ N/m, $\Delta P=10^{7}$ Pa and λ=5; (d) ΔP at $\sigma_{dc}=0.03$ N/m, λ=5 and β=2.5 (Symbols are simulation data and lines are fitted model. The vertical line denotes $\tau_{B}$).

Deformation and fragmentation of an oil droplet with time was characterised using the variation of perimeter, P with time. The influence of various parameters on perimeter normalised by the initial perimeter $\left(P_{0}\right)$ is shown in in Fig. 9. The droplet perimeter was quantified by extracting the oil drop edge at different time steps. The temporal evolution of the dimensionless perimeter $\left(P/P_{0}\right)$ of an oil droplet under different interfacial tension values $\left(\sigma_{dc}=0.01,\;0.03,\;0.05\right)$ during a cavity collapse event, where the droplet is subjected to intense inertial and pressure-driven forces, is presented in Fig. 9a. Droplets with lower interfacial tension $\left(\sigma_{dc}=0.01\right)$ exhibit greater deformation, with the $P/P_{0}$ rising by nearly two orders of magnitude, highlighting their susceptibility to instabilities and reduced resistance to shape changes. Conversely, higher surface tension $\left(\sigma_{dc}=0.05\right)$ provides a stronger restoring force that limits interfacial growth and deformation. This trend underscores the critical role of interfacial tension in modulating droplet dynamics during cavity collapse, where lower $\sigma_{dc}$ enables enhanced instability growth and larger $P/P_{0}$ expansion, while higher $\sigma_{dc}$ promotes more stable and compact interface evolution.

The influence of viscosity ratio $\left(\lambda \right)$ on the evolution of $P/P_{0}$ is shown in Fig. 9b. For a low viscosity ratio (λ=1), the droplet is more susceptible to deformation under the collapsing cavity-induced flow, resulting in increased interfacial stretching and larger dimensionless perimeters. Intermediate viscosity ratio (λ=100) shows moderate deformation but with noticeable scatter in data, indicating intermittent interfacial instabilities possibly due to complex vortex-droplet interactions. In contrast, at high viscosity ratio (λ=500), the droplet retains its shape, with the dimensionless perimeter remaining nearly constant throughout the collapse, demonstrating strong internal viscous resistance to deformation. This behaviour highlights the critical damping role of internal viscosity; higher droplet viscosity suppresses interface distortion and resists elongation under external stress, thereby stabilizing the droplet morphology during the dynamic collapse process. The simulated evolution $P/P_{0}\;$ for different values of β (0.075,2.5,5) is illustrated in Fig. 9c. For β=0.075, droplet exhibit no deformation, and the $P/P_{0}$ does not change as the interfacial tension of the droplet can withstand the driving pressure of the cavities owing to its small size. Conversely, at higher (=5), restoring force is weaker and thus leads greater deformation. This trend exhibits the critical role of size ratio in modulating droplet dynamics during cavity collapse, where higher β enables enhanced instability growth and larger perimeter expansion, while lower β illustrates no drop breakage. The temporal evolution of $P/P_{0}$ for different driving pressure (ΔP) is shown in Fig. 9d. For low driving pressure $\left(\Delta P=1\;x\;10^{6}\;Pa\right)$, the droplet exhibits significant deformation and tends to wrap around the collapsing cavity, leading to an increase in dimensionless perimeter without causing breakup. The perimeter subsequently decreases during cavity collapse due to retraction. Intermediate driving pressure $\left(\Delta P=5\;x\;10^{6}\;Pa\right)$, the droplet undergoes more pronounced deformation with a clear rise in dimensionless perimeter, and the fragmentation of the droplet also takes place, leading to a minimal decrease in perimeter with time. At high driving pressure $\left(\Delta P=1\;x\;10^{7}\;Pa\right)$, the $P/P_{0}$ increased exponentially, representing drop deformation and leading to fragmentation.

The simulated results indicate that the perimeter scales exponentially with time (t) as:

(24) $$P=P_{0}\;e^{k\;t}$$

**Fig. 10:**
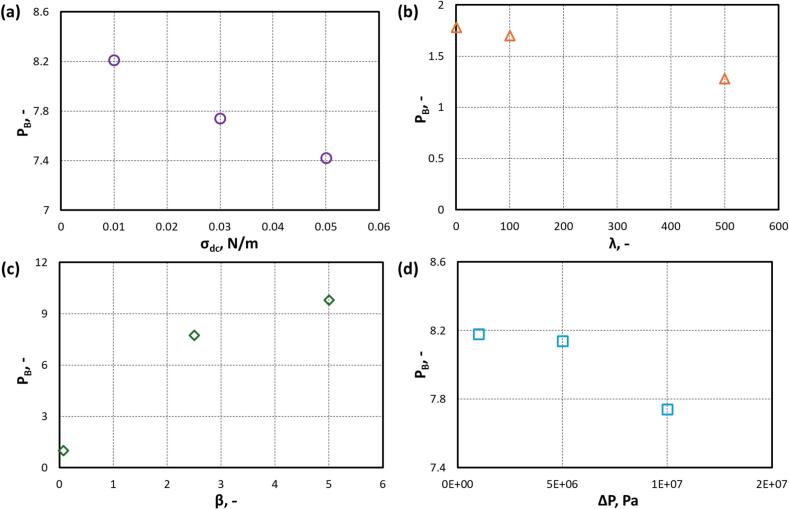
Perimeter of droplet at breakup $\left(P_{B}\right)$ with (a) $\sigma_{dc}$; (b) λ; (c) β and (d) ΔP

Where k is the exponential rate constant $\left(\mu s^{-1}\right)$. The fitted values of k are listed in Table 4. Using the simulated results, a critical value of dimensioless perimeter where droplet breaks ($P_{B}$) and corresponding droplet breakup time ($\tau_{B}$) were identified. The $\tau_{B}$ was measured from the pont at which the cavity begins to expand. The values of $\tau_{B}$ are shown in Fig. 9 by vertical dash lines. Fig. 9a has one breakup time for all the $\sigma_{dc}$ cases, indicating that interfacial tension does not significantly influence $\tau_{B}$. The value of $\tau_{B}$ is influenced by λ, β and ΔP as shown in Fig. 9b and c. The values of $P_{B}$ and $\tau_{B}$ for all the simulated cases are listed in Table 4. In order to convey observed trends, the influence of four key parameters on $P_{B}$ is shown in Fig. 10. It can be seen from Fig. 10, $P_{B}$ decreases with an increase in $\sigma_{dc}$, λ and ΔP. Whereas, $P_{B}$ increases with increase in β.

**Table 4:** Values of $P_{B}$, k and $\tau_{B}$ for key parameters.

**S. No.**	**Parameter**	**Value**	$P_{B},\;-$	k**,**$\mu s^{-1}$	$\tau_{B}$**,**μs
1	$\sigma_{dc}$(N/m)	0.01	8.21	0.13	14
		0.03	7.74	0.12	14
		0.05	7.42	0.11	14
2	λ(−)	1	1.78	0.04	14
		100	1.70	0.033	16
		500	1.28	0.005	26.6
3	β(−)	0.075	−	0	∞
		2.5	7.74	0.04	14
		5	9.8	0.1	15
4	ΔP(Pa)	$1\;x\;10^{6}$	8.18	0.08	26.6
		$5\;x\;10^{6}$	8.14	0.12	15
		$1\;x\;10^{7}$	7.74	0.12	14

### 4.8 Energy dissipation rate(ε)

**Fig. 11:**
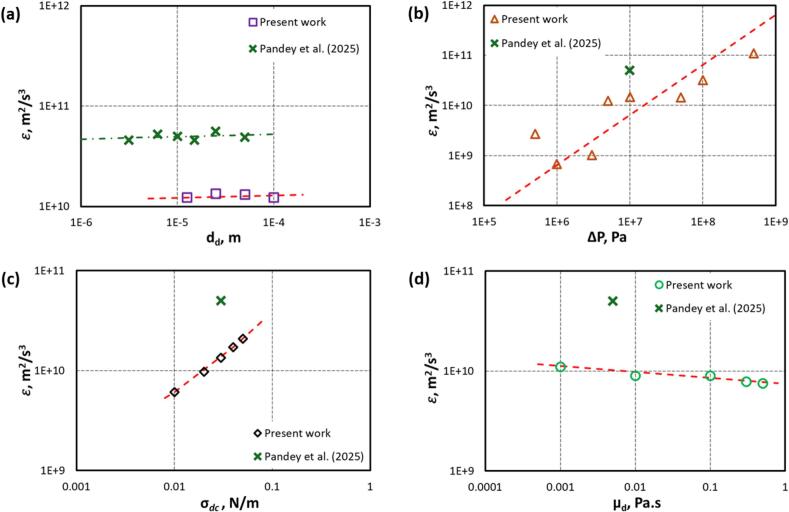
Influence of key parameters on energy dissipation rate (a) $d_{d}$; (b) ΔP; (c) $\sigma_{dc}$ and (d) $\mu_{d}$ (Symbol represents simulation data and dotted line represents fitted data).

Key parameters influencing cavity-droplet interactions are relative sizes of cavity and droplet (β), distance between cavity and droplet (γ), driving pressure (ΔP) and physical properties like interfacial tension between droplet and continuous phase $\left(\sigma_{dc}\right)$ and viscosity of droplet phase $\left(\mu_{d}\right)$. The influence of the distance between cavity and droplet (γ) for the case of a single cavity was investigated in our earlier work [10] and was not included in the present work. In this section, the influence of other parameters on ε generated by collapsing cavities in presence of a nearby oil droplet is discussed. The results obtained for a single cavity-droplet interactions from the previous work of Pandey et al. [10] are also included in Fig. 11a–d as a reference.

The influence of the $d_{d}$ (in other words, β) on ε is shown by Fig. 11a. The results indicate that larger droplet led to a marginal increase in ε when subjected to equivalent ΔP. This can be attributed to enhanced exposure to the pressure and velocity fields induced by cavity expansion. The influence of $d_{d}$ was found to be rather weak $\left(\propto \;d_{d}^{0.03}\right)$. A comparison with Pandey et al. [10] shows that while both studies exhibit the same trend ($\propto \;d_{d}^{0.03}\;or\;\beta^{0.03})$. In the present configuration involving two cavities with a droplet positioned between them, the value of ε is reduced compared to the single cavity considered previously [10] due to the redistribution of ε around the droplet as a result of the collapsing cavities. Conversely, in the case of a single cavity–droplet interaction, ε is higher because of the asymmetric nature of collapse. The variation of ε with ΔP (corresponding to cavitation driving pressure or intensity) is illustrated by Fig. 11b. The results show an almost linear increasing trend of ε with increasing P $\left(\propto {\Delta P}^{0.9}\right)$. As the ΔP increases, the intensity of cavity implosion rises significantly, leading to stronger inertial flows, elevated shear stresses around the droplet, and enhanced interfacial instabilities. Fig. 11b, does not exhibit any signs of saturation in ε even at higher values of ΔP, suggesting that the dissipation mechanisms remain highly responsive to increasing driving forces throughout the explored pressure range. Fig. 11c shows the influence of the interfacial tension, $\left(\sigma_{dc}\right)$​. It can be seen that ε increases linearly with the increase in interfacial tension. The influence of $\mu_{d}$ on energy dissipation rate is seen in Fig. 11d. The energy dissipation rate is not significantly influenced by the value of $\mu_{d}\;$ $\left(\propto \;\mu_{d}^{-0.06}\right)$ for the entire explored range. Since the ε does not change significantly with a change in $\mu_{d}$, the deformation of a droplet with higher $\mu_{d}$ is expected to be lower (refer to Fig. 9).

For establishing a correlation to estimate the influence of key parameters on generated energy dissipation rate, a non-dimensional energy dissipation rate $\left(\varepsilon^{*}\right)$ is defined as:

(25) $$\varepsilon^{*}=\frac{\varepsilon }{\varepsilon_{o}}\;\;where\;\varepsilon_{o}=\frac{{\left(\Delta P/\rho_{\text{c}\;}\right)}^{3/2}}{R_{cav}}$$

Where $\varepsilon_{o}$ represents the energy dissipation rate generated by the collapse of a single cavity in an infinite fluid domain without the presence of any object in its vicinity. The simulated results can be represented by the following correlation:

(26) $$\varepsilon^{*}=C\left(\frac{\sigma_{dc}}{\sigma_{cav-c}}\right){\left(\frac{\Delta P}{P_{\infty }}\right)}^{-0.6}\beta^{0.03}\;\lambda^{-0.06}$$

**Fig. 12:**
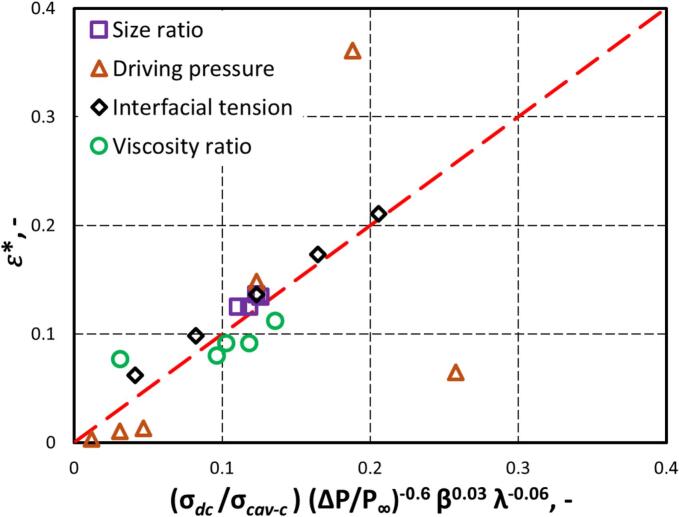
Variation of $\varepsilon^{*}$ with $\left(\frac{\sigma_{dc}}{\sigma_{cav-c}}\right){\left(\frac{\Delta P}{P_{\infty }}\right)}^{-0.6}\beta^{0.03}\;\lambda^{-0.06}$ (Symbol represents simulation data, and dotted line represents fitted data).

Where the value of C was found to be 5 (see Fig. 12). The maximum value of $\varepsilon^{*}$ was found to be 0.26, suggesting that up to 26% of the cavity’s collapse energy is dissipated during the first collapse. This partial energy dissipation explains why cavities undergo multiple expansion–compression cycles. A value of $\varepsilon^{*}=1$ is possible only when total energy is dissipated during the first collapse, a condition not observed in the present simulations. The simulated values of energy dissipation rate $\left(\varepsilon^{*}\right)$ may also be related to ${We}_{dc}$ and $Oh_{dc}$ as:

(27) $$\varepsilon^{*}=C_{{We}_{dc}=1}\;\;{{We}_{dc}}^{-0.9}$$

(28) $$\varepsilon^{*}=C_{{Oh}_{dc}=1}\;{{Oh}_{dc}}^{-0.1}$$

Where, ${We}_{\text{dc}\;}$ is the Weber number (Equation (7) for the droplet and continuous phase, ${Oh}_{dc}$​ is the Ohnesorge number (Equation (5) for the droplet and continuous phase. $C_{{We}_{dc}=1}$ and $C_{{Oh}_{dc}=1}$ are the values of $\varepsilon^{*}$ at ${We}_{\text{dc}}=1$ and ${Oh}_{dc}=1$, respectively.

**Fig. 13:**
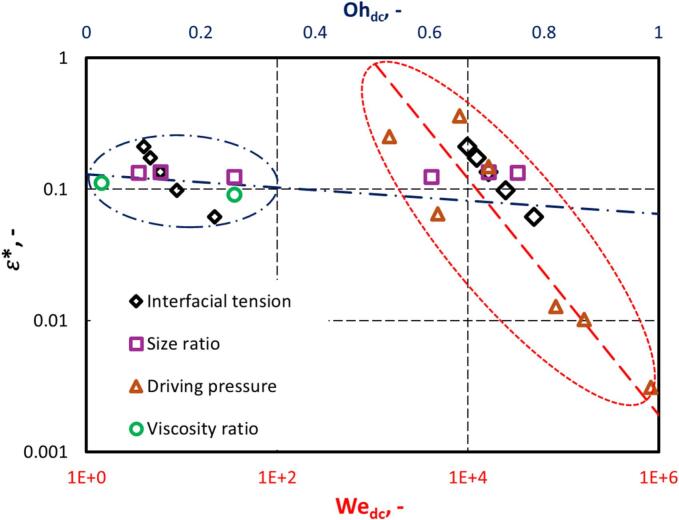
Variation of $\varepsilon^{*}$ with ${We}_{dc}$ and ${Oh}_{dc}$ (Symbol represents simulation data, and dotted line represents fitted data. Red and blue colour ovals include key parameters used in the calculation of ${We}_{dc}$ and ${Oh}_{dc}$). (For interpretation of the references to color in this figure legend, the reader is referred to the web version of this article.)

$\varepsilon^{*}$ as functions of the ${We}_{\text{dc}\;}$ and ${Oh}_{dc}$ provide insight into the dominant physical mechanisms governing droplet–cavity interactions under varying fluid dynamic conditions (as shown in Fig. 13). $\varepsilon^{*}$ exhibits a steep decline with increasing ${We}_{\text{dc}\;}$ [Equation (27)]. A high ${We}_{\text{dc}\;}$ implies that inertial effects strongly dominate over surface tension. Consequently, as ${We}_{\text{dc}\;}$ increases, the cohesive interfacial forces become insufficient to sustain droplet integrity under strong inertial stresses, leading to early fragmentation, spreading, or damped deformation, which results in a sharp drop in the energy dissipation rates. This behaviour reflects the diminishing role of interfacial cohesion in governing energy transfer. In contrast, the variation of $\varepsilon^{*}$ with the ${Oh}_{dc}$ reveals a much weaker declining trend $\left(\propto Oh_{dc}^{-0.1}\right)$. The minimal sensitivity of ${Oh}_{dc}$ implies that within the examined range, viscous effects exert only a secondary influence on the droplet deformation. While increased viscosity (higher ${Oh}_{dc}$) can resist deformation and dissipate energy more diffusely; this damping effect progresses gradually. This suggests that although viscosity plays a role in modulating droplet response, it does not drastically alter the energy transmission dynamics unless extremely high viscosities are involved.

## 5 Conclusions

Present study investigates the drop breakage via two collapsing cavities using the multi-fluid VOF method. Axisymmetric simulations were performed in a cylindrical domain where dispersed phases, cavity, and oil droplet are suspended in water. The drop breakage under various conditions, such as interfacial tension, viscosity of droplet, size ratio and driving pressure, were studied. The primary objectives are to understand the physics behind the droplet breakage by collapsing cavities and quantify the influences of key parameters such a viscosity, interfacial tension, size ratios and driving pressure on the droplet deformation and fragmentation. An attempt was made to quantify the energy dissipation rates generated by two collapsing cavities while interacting with an oil drop present in the vicinity. The key conclusions are:

• Energy dissipation rate (ε) increases linearly with the interfacial tension $\left(\propto \sigma_{dc}\right)$ and almost linearly with driving pressure $\left(\propto \Delta P^{0.9}\right)$.
• Influence of drop size $\left(\propto \beta^{0.03}\right)$ and viscosity $\left(\propto \lambda^{-0.06}\right)$ on energy dissipation rate is weak.
• Dimensionless perimeter $\left(P/P_{0}\right)$ scales exponentially with time $\left(\propto e^{kt}\right)$.
• Dimensionless perimeter of droplet at breakup $\left(P_{B}\right)$ decreases with an increase in interfacial tension, viscosity, driving pressure and increases with an increase in size ratio.
• Non-dimensional energy dissipation rate $\left(\varepsilon^{*}\right)$ is weakly dependent on ${Oh}_{\text{dc}\;}$($\propto {{Oh}_{\text{dc}}}^{-0.1}$) and almost linearly dependent on ${We}_{\text{dc}\;}$($\propto {{We}_{\text{dc}}}^{-0.9}$).

These findings provide mechanistic insights into droplet deformation in the presence of two collapsing cavities. The presented results would provide a sound basis for further work on developing quantitative models of droplet breakup occurring in cavitation-assisted emulsification.

## 6 Data availability statement

The data supporting this study's findings are available from the corresponding author upon reasonable request.

## CRediT authorship contribution statement

**Deepak K. Pandey:** Writing – original draft, Visualization, Software, Methodology, Investigation, Formal analysis. **Rupak Kumar:** Writing – original draft, Visualization, Software, Methodology, Investigation, Formal analysis. **Vivek V. Ranade:** Writing – review & editing, Supervision, Project administration, Methodology, Funding acquisition, Conceptualization.

## Declaration of competing interest

The authors declare that they have no known competing financial interests or personal relationships that could have appeared to influence the work reported in this paper.
