# Supplementary material for: Dynamics of droplet breakup symmetrically placed between two collapsing cavities via numerical simulations
Source: Ultrason Sonochem. 2025 Aug 5;120:107493. doi: 10.1016/j.ultsonch.2025.107493 (PMC12345313; doi:10.1016/j.ultsonch.2025.107493)
Supplement: Supplementary Data 1 [file mmc1.pdf]

SUPPLEMENTARY MATERIAL

Dynamics of Droplet Breakup Symmetrically placed between Two Collapsing Cavities via Numerical Simulations

Deepak K. Pandey+, Rupak Kumar+ and Vivek V. Ranade*
Multiphase Reactors and Intensification Group
Bernal Institute, University of Limerick, Limerick V94T9PX, Ireland
+ Equal contributions; *Email: vivek.ranade@ul.ie

S1. Validation

The numerical model is qualitatively validated against the experimental observations reported by Luo and Niu [53], as shown in Figure 3, to assess whether the simulation can accurately capture the dynamics of interacting cavities. This particular study was selected for comparison because the current work also involves the interaction of two cavities with a droplet. Luo and Niu [53] did not report the initial cavity radius in their study. They only reported that the initial separation between the two cavities at inception was 1.23 mm, and the maximum cavity size was reported as 830 μm at 112.5 μs. We formulated our simulation case using this reported information. The initial radius of the cavity was estimated using the relation $R_{initial} = R_{max}/6.25$, as proposed by Orthaber et al. [44]. Since the initial pressure inside the cavities was not specified in the experimental study, we adopted an approach consistent with our previous work [10], where the initial pressure was varied to approximate the conditions of laser-induced cavitation. Based on that methodology, the initial cavity pressure in the current simulations was taken as 10^7 Pa. Because of the uncertainties involved in estimating the initial conditions for our simulations, there may be some differences in the simulated and experimental results.

An axisymmetric computational domain is employed in this study, as shown in Figure S1. The domain represents a portion of a large cylindrical space filled with a continuous phase (water), where two cavities (air) of identical size and pressure difference are positioned 1.23 mm apart. The size of the computational domain is chosen to be 100 times the maximum radius of the cavities (100 x 830 mm) to minimize the influence of boundary conditions on the interaction between the cavities and droplets. Pressure outlet and wave-transmissive boundary conditions are applied at the outlets to simulate a larger fluid domain. Initially, the entire domain is at atmospheric pressure, except for the cavities. After conducting the grid independence test, the minimum grid size used in the simulation was 0.3 μm, ensuring sufficient spatial resolution to capture small-scale features associated with cavity evolution and droplet fragmentation. The computational model used is the same as the one discussed in Section 3 of the manuscript.

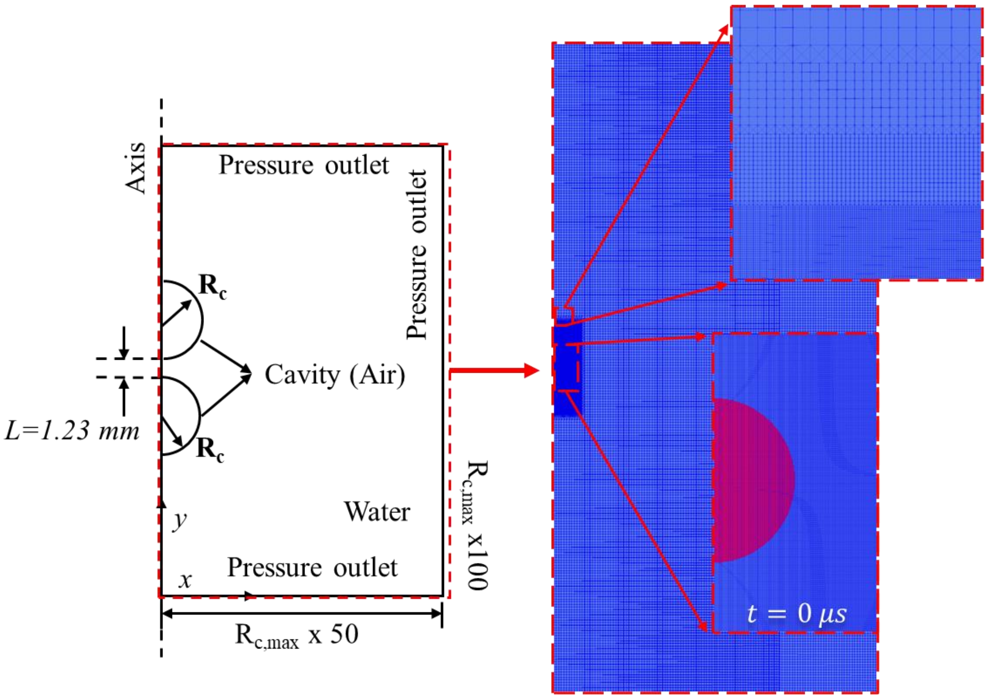

Figure S1: Computational domain and grid distribution used for the validation

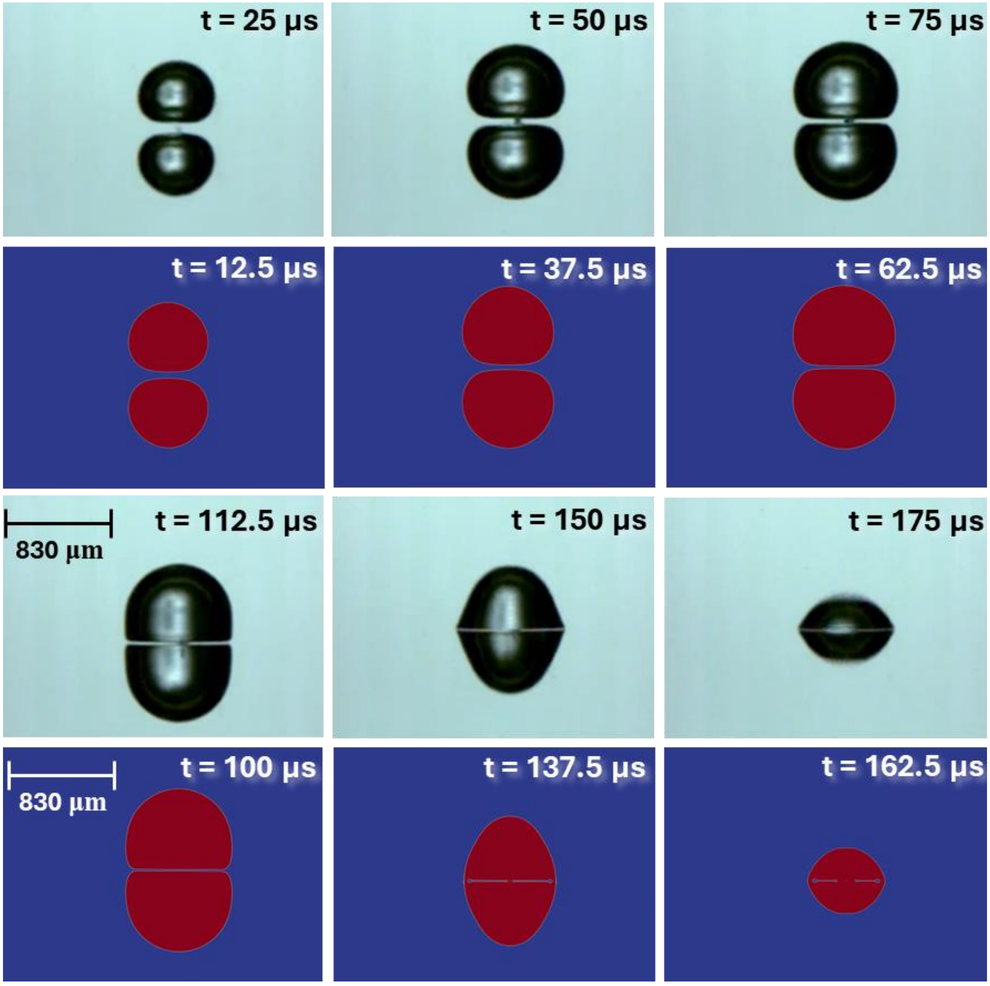

Figure 3: Interactions of two cavitation bubbles generated in water (Top: Luo and Niu [53] and Bottom: Present solver validation)

S2. Effect of gravity

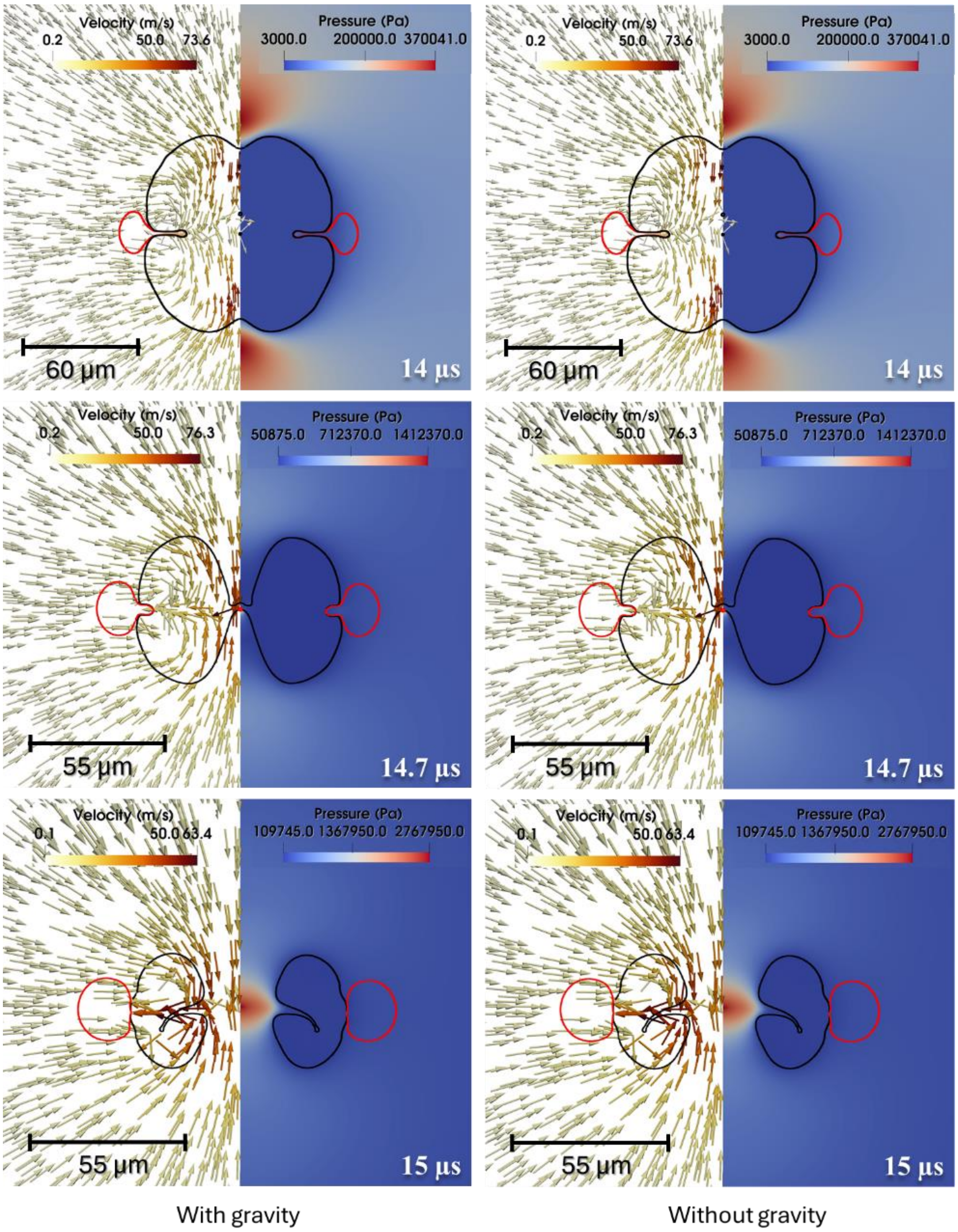

Figure S2: Effect of gravitational force on cavities-droplet interaction for $\sigma_{dc} = 0.05$ N/m at $\mu_d = 0.005$ Pa.s and $\beta = 2.5$

S3. Drop pinch-off

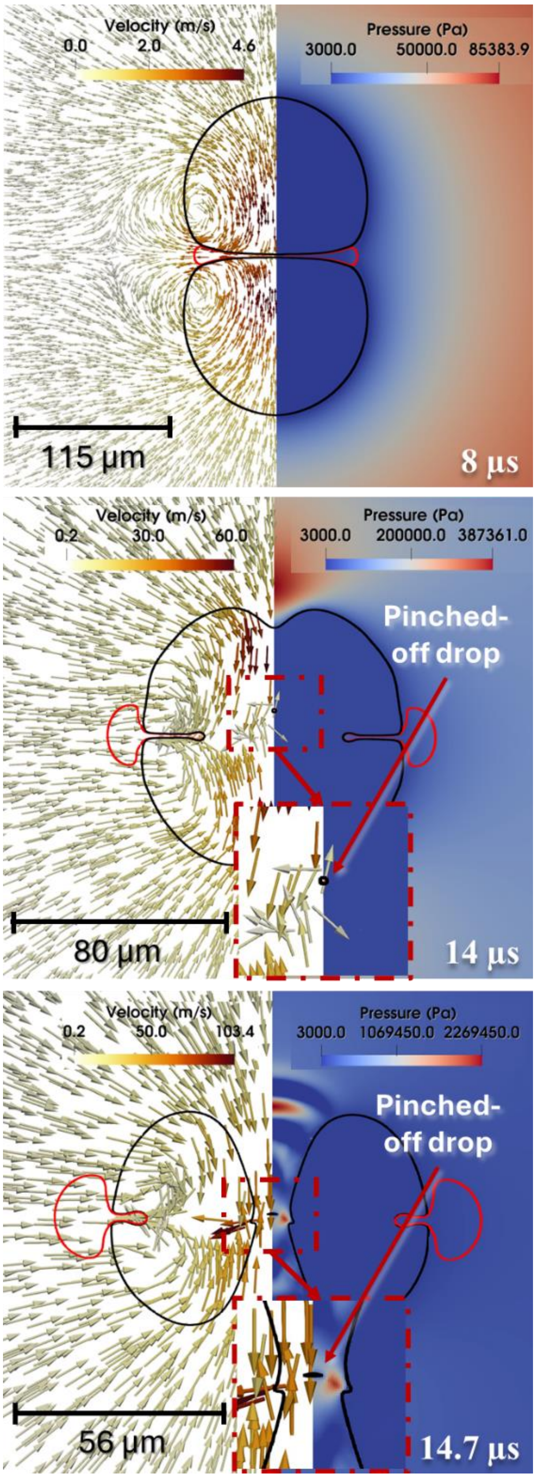

Figure S3: Pinch-off drop creating instability
